# A Novel ceRNA Axis LOC121818100/Novel‐miR‐400/SSRP1 Regulated Muscle Growth and Injury Repair in Sheep

**DOI:** 10.1002/jcsm.13836

**Published:** 2025-06-05

**Authors:** Fan Yang, Runqing Chi, Runan Zhang, Kai Liu, Pingqing Wang, Ran Di, Xiaoyun He, Xiangyu Wang, Yufang Liu, Mingxing Chu

**Affiliations:** ^1^ State Key Laboratory of Animal Biotech Breeding Institute of Animal Science, Chinese Academy of Agricultural Sciences (CAAS) Beijing China; ^2^ College of Bioengineering Chongqing University Chongqing China

**Keywords:** ceRNA axis, CTX animal model, muscle injury repair, skeletal muscle myoblast proliferation, SSRP1

## Abstract

**Background:**

In mammals, skeletal muscle growth is a delicate process. The construction of competitive endogenous RNA (ceRNA) networks provides an effective way to analyse the molecular mechanism of complex trait formation. The aim of this study was to investigate the role of the ceRNA axis in skeletal muscle development.

**Methods:**

Transcriptome sequencing (RNA‐seq) technology was used to analyse the RNA expression profile of longissimus dorsi muscle of different breeds of sheep. MiRanda and qTar databases were used to construct the ceRNA network. The key ceRNA regulatory axis was screened by bioinformatics analysis. Using primary sheep myoblast (SMs) and mouse models, we evaluated the effects of target gene structure specific recognition protein 1 (SSRP1) and related noncoding RNA (ncRNA) on cell proliferation and muscle development in vitro and in vivo functional experiments. Dual‐luciferase reporter assay, fluorescence in situ hybridization (FISH), RNA immunoprecipitation (RIP) and RNA pull‐down assay were used for mechanistic analysis.

**Results:**

The number of differentially expressed (DE) lncRNA, miRNA and mRNA in STH and SNT sheep (*n* = 5) longissimus dorsi muscle was 99, 53 and 1864, respectively (*p* < 0.05). A ceRNA network containing 146 lncRNA‐miRNA‐mRNA axes was constructed (*p* < 0.05). Bioinformatics analysis showed that LOC121818100/Novel‐miR‐400/SSRP1 was most correlated with skeletal muscle development (*p* < 0.05). Analysis in SMs showed that the expression of proliferation markers (CCND1, CCNE1, PCNA and PAX7) was decreased (−30%, *p* < 0.05) after inhibition of LOC121818100 or SSRP1 expression. Overexpression of Novel‐miR‐400 decreased the expression of proliferation markers (−80%, *p* < 0.01). SSRP1 knockdown decreased the proliferation activity of SMs (−80%, *p* < 0.05). After SSRP1 knockdown, the proportion of SMs entering S phase decreased (−3.22%, *p* < 0.05). 5‐ethynyl‐2′‐deoxyuridine (EdU) analysis showed the same trend (*p* < 0.05). The results of mouse model also confirmed that SSRP1 was positively correlated with skeletal muscle development (*p* < 0.05). It was confirmed that LOC121818100 acts as a sponge for Novel‐miR‐400 and reduces its inhibitory effect on SSRP1. This interaction promotes skeletal muscle development in sheep.

**Conclusion:**

Our results identified a role for SSRP1 in promoting skeletal muscle growth. Sheep skeletal muscle development is regulated by LOC121818100/Novel‐miR‐400/SSRP1 axis. These findings provide new insights into the role of the ceRNA machinery in regulating skeletal muscle growth.

## 1 Introduction

Skeletal muscle refers to the muscle attached to the bone and is a type of striated muscle. In mammals, skeletal muscle can account for up to 30% of body weight [1]. In animal husbandry, the growth of skeletal muscle directly affects the yield and quality of meat [2, 3]. In addition, skeletal muscle dysfunction can lead to various muscle diseases, including muscle hypertrophy, atrophy and tumours [4, 5]. Therefore, understanding the regulatory mechanisms of skeletal muscle development could help treat diseases related to human muscles and improve meat production in agricultural animals. However, muscle growth is a refined, multistep process involving multiple biomolecules. To date, the mechanisms of muscle formation have remained largely unknown.

In recent years, attention has been paid to the role of ncRNA in skeletal muscle development [6]. Long noncoding RNA (lncRNA), a class of endogenous ncRNA found in eukaryotes, was once considered to be genetic ‘transcription noise’ [7]. However, with the development of RNA research, it has been found that lncRNA can regulate gene expression through multiple mechanisms [8, 9]. Among them, some lncRNA play the role of sponge and compete with mRNA to bind microRNA (miRNA) in the cytoplasm, which is defined as ceRNA mechanism [10]. It is worth noting that studies have reported the role of lncRNA in muscle development. For example, in mouse myoblasts, H19 acts as a molecular sponge for let‐7, effectively blocking the inhibitory effect of let‐7 on high‐mobility histone A2 (HmgA2) and Dicer. This inhibition further affects the differentiation process of skeletal muscle [11]. In chickens, lncIRS1, as an adsorbent of miR‐15, can regulate the expression of IRS1, thereby promoting the generation of skeletal muscle [12]. In addition, LCC‐ORA induces muscle atrophy by adsorbing miR‐532‐3p and interacting with IGF2BP2, while inhibiting skeletal muscle generation in mice [13]. Together, these studies could provide valuable insights into the ceRNA mechanisms by which lncRNA control muscle growth and regeneration. However, particularly in sheep, the involvement of lncRNA in this complex process remains relatively unexplored.

In this study, RNA expression profiles of small tail Han sheep (STH) with fast muscle growth and Sunit sheep (SNT) with slow muscle growth were determined by RNA‐seq. Then, a ceRNA network composed of lncRNA, miRNA and mRNA was constructed. Gene Ontology (GO) and the Kyoto Encyclopedia of Genes and Genomes (KEGG) analysed mRNA in the ceRNA network to screen out ceRNA regulatory pathways (LOC121818100/Novel‐miR‐400/SSRP1) that are closely related to skeletal muscle growth in sheep. In vitro experiments demonstrated that LOC121818100, as a Novel‐miR‐400 sponge, affects the expression of SSRP1, ultimately regulates the growth of SMs and affects the development of skeletal muscle in sheep. In addition, in vivo experiments in mice further confirmed the role of SSRP1 in skeletal muscle growth and regeneration. This suggests that SSRP1 may have a conservative role in mammalian skeletal muscle development, although the regulatory mechanisms may be different. However, it also provides a vivid example that conservation of function does not equal conservation of regulation. All in all, our findings provide new insights and targets for meat sheep breeding and the treatment of muscle diseases.

## 2 Material and Methods

### 2.1 Experimental Animals and RNA Extraction

Female SNT and STH (*n* = 5) at 2 months old were raised in captivity under restricted feeding conditions (the same volume of feed). The lambs were slaughtered at the age of 8 months and weighed. The average carcass weight was 33.88 ± 4.54 kg in STH group and 22.50 ± 1.79 kg in SNT group (*p* < 0.05). Total RNA was isolated from cells and tissue using TRIzol reagent (TIANGEN, China). RNA was isolated from the nucleus and cytoplasm of cells using a Cytoplasmic & Nuclear RNA Purification Kit (biomars‐technology, China).

### 2.2 RNA‐Seq

On the basis of obtaining RNA samples, sequencing experiments were carried out. The specific steps include library construction, library detection and computer sequencing. The library construction and sequencing in this study were completed by Wuhan Fraser Gene Information Co., Ltd.

### 2.3 Constructing ceRNA Network

miRanda software and qTar software were used to predict target genes, and the results were intersected. For the obtained miRNA and lncRNA, miRNA and mRNA relationship pairs, the correlation coefficient was calculated by Pearson correlation analysis, and the targeted relationship pairs with significant negative correlation were screened. Based on the ceRNA theory, mRNA and lncRNA coregulated by miRNA were sorted out, and the regulatory network was visualized using Cytoscape (3.8.0).

### 2.4 GO and KEGG Analyses

GO enrichment analysis method is hyper‐geometric distribution, which is the same as KEGG enrichment analysis method. We selected GO term with FDR ≤ 0.05 as the significantly enriched GO entries. The pathway with a *Q* value of < 0.05 was defined as the significantly enriched KEGG pathway. Enrichment operations were completed using the online site https://www.bioinformatics.com.cn/to.

### 2.5 RT‐qPCR

RT‐qPCR uses RNA reverse transcription cDNA from sheep tissues or cells as a template. RT‐qPCR was performed using TaKaRa SYBR premixed Ex Taq II kit (TaKaRa, Japan). Follow the manufacturer's instructions. All primers were synthesized by Sangon (Shanghai, China), and the sequence of primers was shown in Table S1.

### 2.6 Western Blot Assay

Total protein was extracted using the total protein extraction kit (Solebro, China). Protein concentration was determined using the BCA protein concentration assay kit (Solebro, China). The denatured proteins were isolated using the common Western Blot procedure. Analysis was performed by transfer of 10% SDS‐PAGE to PVDF membrane. The antibodies used in this study are as follows: anti‐SSRP1 (1:1000; Proteintech, Wuhan, China); anti‐PAX7 (1:1000; Abcam, Cambridge, England); and anti‐GAPDH, anti‐PCNA, anti‐CCND1, anti‐CCNE1 and anti‐β‐tubulin (1:500; Bioss, Beijing, China). The complete image of the western blot results involved in this paper is shown in Figure S1.

### 2.7 Immunohistochemistry (IHC) Assay

Five‐micrometre‐thick paraffin sections were made from the longest muscle tissue of the sheep back. After hydration in xylene and a series of graded ethanol solutions, endogenous peroxidase was blocked with 3% hydrogen peroxide. The slice samples were blocked with 5% bovine serum albumin (BSA) for 1 hour, and then treated with anti‐SSRP1 (1:1000; Proteintech, Wuhan, China) overnight at 4°C. After washing with PBS, the samples were incubated for 1 h with anti‐IgG secondary biotin conjugate antibody (Beyotime, Shanghai, China). Next, they were treated with 0.02% avidin‐biotin‐peroxidase complex (Solarbio, Beijing, China) and 40% 3,3′‐diaminobenzidine (2 μg/mL) for 1–10 min. Finally, the slides were reverse‐stained with haematoxylin and observed under a light microscope (Leica, Germany).

### 2.8 Cell Culture and Identification

As described in previous studies [14], SMs was isolated from the foetal state longissimus muscle of sheep. The isolated SMs was identified by immunofluorescence, and its differentiation ability was analysed (Figure S2). SMs were cultured in high glucose medium (DMEM) containing 10% foetal bovine serum (FBS, Gibco) and 1% penicillin–streptomycin (Solarbio, Beijing, China). The culture conditions of HEK293T cell line were consistent with SMs. When SMs grew to 90%, the differentiation medium (replacing 10% FBS with 2% horse serum) was changed to induce SMs differentiation. All cell lines were maintained in a humidified incubator set at 37°C with 5% CO_2_.

### 2.9 siRNAs, Vector Construction and Transfection

Before transfection, SMs were cultured in six‐well plates until the fusion reached 70%–80%. Transfection procedures follow the GenePharma's instructions (Protocol Pub. No. MAN0007824 rev. 1.0). The overexpressed vector was pcDNA‐3.1. All plasmids and oligonucleotides present in this study were constructed by GenePharma (Shanghai, China). In vivo transfection of animals is performed using the cotransfection reagent in vivo‐jetPEI (Noninbio, Shanghai, China), according to the manufacturer's instructions. To put it simply, a certain amount of nucleic acid and in vivo‐jetPEI is diluted separately into a glucose solution and then mixed to form a complex for intramuscular injection.

### 2.10 CCK8 Assay

SMs were grown in 96‐well plates, and the assessment of cell proliferation rates occurred at 0, 6, 12, 24 and 48 h after transfection, utilizing the CCK8 Cell Proliferation Assay Kit (Solebio, Beijing, China). The optional density (OD) was measured at 450 nm using a microplate reader (Thermo, United States).

### 2.11 EdU Assay

SM proliferation status was determined using the CBeyoClick EdU‐488 Cell proliferation assay kit (Beyotime, Shanghai, China) according to the manufacturer's instructions.

### 2.12 Cell Cycle Assay

In six‐well plates, SMs were transfected at a density of 2 × 10^6^, and cells were harvested 36 h after transfection. They were washed twice with cold PBS and fixed with 70% ethanol at 4°C overnight. Afterward, the cells were washed twice again to remove any residual ethanol. Next, 100 μL of RNase A (Vazyme, Nanjing, China) was added and incubated at 37°C for 30 min. Following this, the cells were stained with 400 μL of propidium iodide (Beyotime, Shanghai, China) for 30 min. Flow cytometry (FACS) was performed to evaluate the cells at 488 nm, and ModFit LT (4.1) was used for analysis.

### 2.13 Mouse Model of Muscle Injury

Mice (C57BL/6) were purchased from the Peking University Laboratory Animal Center (Beijing, China). Cardiotoxin (CTX, Solarbio) was diluted to a concentration of 10 μmol/L in sterile saline, and 50 μL of this solution was injected into the right tibialis anterior (TA) muscle of 6‐week‐old mice to induce muscle injury. TA tissue samples were collected at different times after injury and fixed with haematoxylin–eosin (HE) staining.

### 2.14 Dual‐Luciferase Reporter Assay

The luciferase reporter vector was GP‐miRGLO (GenePharma, Shanghai, China). All plasmids were cotransfected with Novel‐miR‐400 mimics or inhibitors. HEK293T cells were harvested 48 h after transfection, and chemiluminescence intensity was measured on a photometer using a dual luciferase reporting system (Vazyme, Nanjing, China), and firefly and Renilla luciferase activity was detected.

### 2.15 FISH

FISH detected the subcellular localization of LOC121818100 and Novel‐miR‐400 in cells. The fluorescent‐labelled LOC121818100 probe and the Novel‐miR‐400 probe were designed and synthesized by GenePharma (Shanghai, China). The instructions for the RNA FISH SA‐Biotin Amplification System kit from GenePharma were followed. Images were collected using a laser confocal microscope (Leica, Germany).

### 2.16 Nucleic Acid Electrophoresis

The cDNA and gDNA PCR products were detected by electrophoresis using 2% agarose gel. The reaction condition was 100 V, and electrophoresis was performed for 30 min. The gel after electrophoresis was photographed using a gel imaging system (Thermo, United States). A complete image of the gel results covered in this paper can be found in Figure S3.

### 2.17 RIP

The RIP test kit (Genecreate, China) is used to detect RIP. Follow the manufacturer's instructions for use.

### 2.18 RNA Pull‐Down Assay

A 3′‐terminal biotinized Novel‐miR‐400 mimics RNA or a negative control (50 nM, GenePharma, Shanghai, China) was transfected into SMs. After 24 h, the cells were washed with PBS, and the cell contents were harvested with a lysate buffer containing 50 U RNase OUT. Biotin‐coupled RNA complexes were collected using the RNA pull‐down kit (Genecreate, China). The magnetic bead‐bound pull‐down RNA was isolated with TRIzol reagent. The abundance of LOC121818100 was analysed by RT‐qPCR.

### 2.19 Statistical Analysis

To determine if two or more groups were statistically significant, the *T* test and one‐way analysis of variance were employed for analysis. Pearson correlation coefficient was used to analyse the correlation. All experiments included at least three biological replicates. *p* < 0.05 was significant.

## 3 Results

### 3.1 ceRNA Networks Were Constructed Based on DE lncRNA, miRNA and mRNA

**FIGURE 1:**
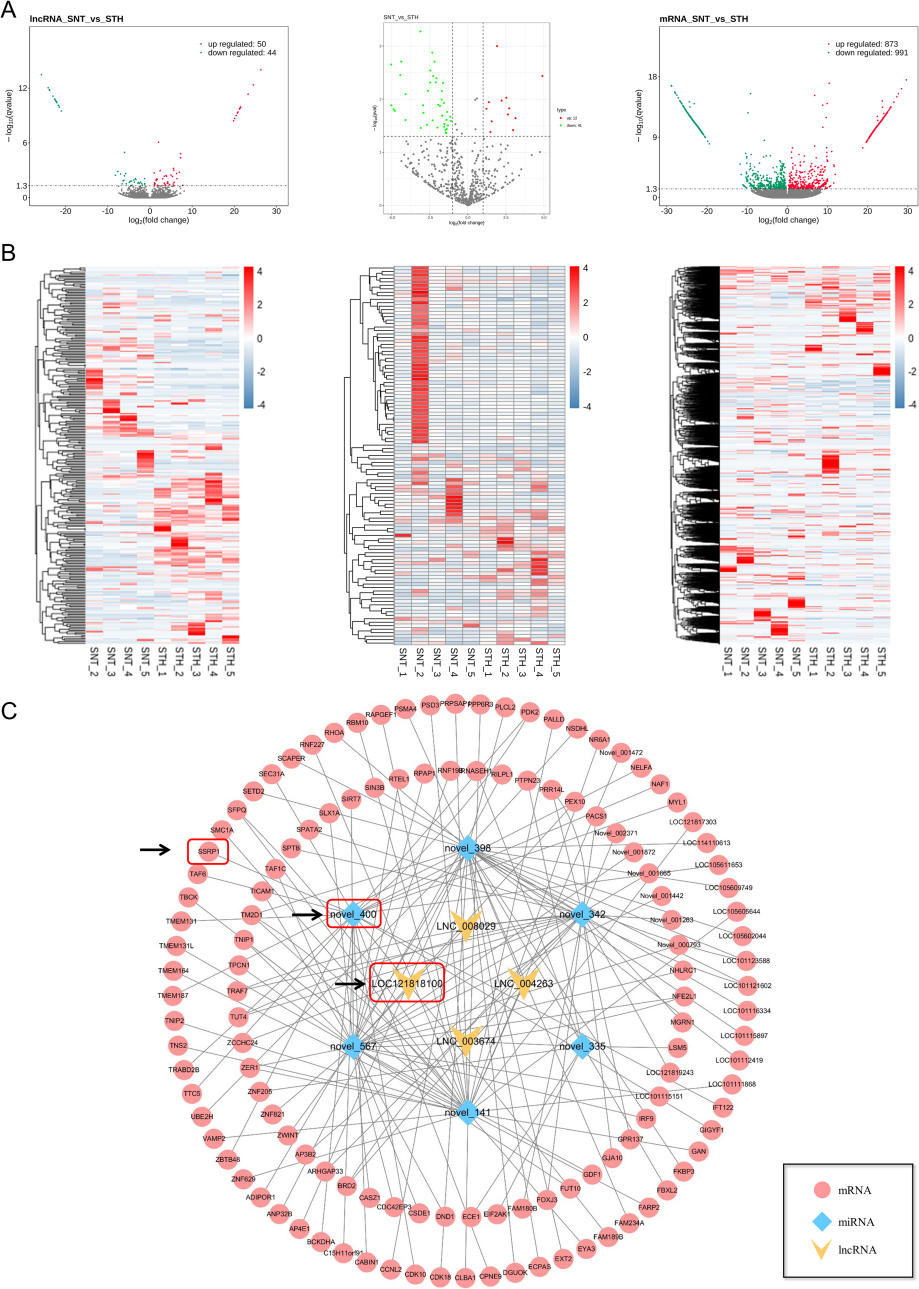
ceRNA networks were constructed based on DE lncRNA, miRNA and mRNA. (A) Volcanic maps of DE lncRNA, miRNA and mRNA between STH and SNT. (B) Heat maps of DE lncRNA, miRNA and mRNA between STH and SNT. (C) Constructed ceRNA networks based on DE RNA and bioinformatics prediction. The red box part for the final selected key molecules.

RNA expression profiles from SNT and STH longissimus dorsi muscles were identified (Figure 1A,B). Compared to the SNT, the STH group had 99 DE lncRNA (50 upregulated and 44 downregulated), 53 DE miRNA (12 upregulated and 41 downregulated) and 1864 DE mRNA (873 upregulated and 991 downregulated). The top 9 DE lncRNA and mRNA can be found in Tables S2 and S3, whereas the top 5 DE miRNA are listed in Table S4.

Following the previously described analysis process (Figure S4), ceRNA interaction network was constructed (Figure 1C). There were 146 lncRNA‐miRNA‐mRNA pathways, including 4 lncRNA, 6 miRNA and 139 mRNA. GO analysis revealed that the DE mRNA within the ceRNA networks are primarily associated with cellular processes (biological process), cellular anatomical entities (cellular component) and binding (molecular function) (Figure S5A). KEGG pathway analysis indicated that these DE mRNA were enriched in 20 pathways. Of them, the endocytosis, tight junction and phagosome signalling pathway were the most enriched (Figure S5B). After checking the functional items enriched by the top 9 DE mRNA (DND1, ARHGAP33, TNS2, PACS1, SSRP1, ZBTB48, RBM10, ANP32B and CSDE1), we found that SSRP1 associated to the most related to GO items of the growth and development, such as metabolism process, cellular process and growth (Figure S5C). In addition, by reviewing UCSU databases (https://genome.ucsc.edu/), it was found that LOC121818100, a lncRNA derived from SSRP1‐related ceRNA axis, is specifically expressed in sarcoplasmic organs, such as muscles, heart and stomach (Figure S5D). Meanwhile, PDLIM3, the parent gene of LOC121818100, was also significantly associated with muscle development [15]. Therefore, this study selected the ceRNA network regulatory axis composed of SSRP1 and its related ncRNAs (LOC121818100/Novel‐miR‐400/SSRP1) as the research object for subsequent experiments (red box shown in Figure 1C).

### 3.2 The Expression Level of LOC121818100/Novel‐miR‐400/SSRP1 in Muscle Tissues

**FIGURE 2:**
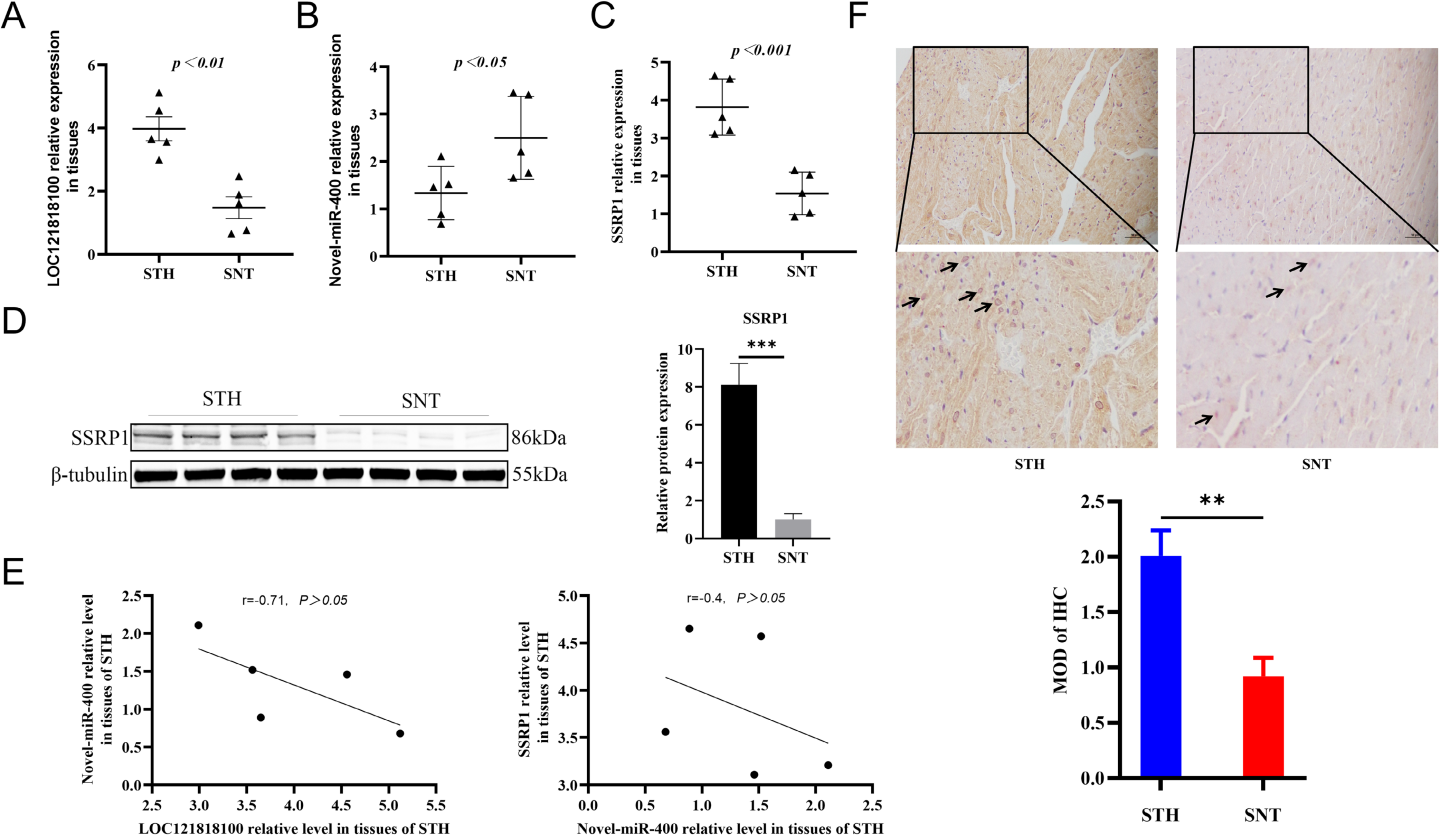
The expression level of LOC121818100/Novel‐miR‐400/SSRP1 in STH and SNT muscle tissues. (A–C) The mRNA expression levels of LOC121818100 (A), Novel‐miR‐400 (B) and SSRP1 (C) in muscle tissues were determined by RT‐qPCR. (D) The protein levels of SSRP1 in muscle tissues detected by Western blot. (E) The correlation between LOC121818100 and Novel‐miR‐400, as well as Novel‐miR‐400 and SSRP1, in muscle tissues. (F) The localization of muscle SSRP1 in STH and SNT was examined by IHC. SNT, sunit sheep; STH, small tailed han sheep. Scale bar, 50 μm. Data are expressed as the mean ± SEM. **p < 0.01, ***p < 0.001.

To confirm the validity of the RNA‐seq data, we examined the expression levels of LOC121818100, Novel‐miR‐400 and SSRP1 in STH and SNT longissimus dorsi muscle tissue. The results showed that mRNA levels of LOC121818100 (Figure 2A) and SSRP1 gene (Figure 2C,D) were higher in fast‐growing STH than SNT. Novel‐miR‐400 expression level was decreased in STH (Figure 2B), which was in contrast with the expression of LOC121818100 and SSRP1 (Figure 2E). IHC assay showed that SSRP1 was widely distributed in sheep muscle tissues, and its expression in STH was higher than SNT (Figure 2F). These results are consistent with RNA‐seq, demonstrating the reliability of the sequencing data and the feasibility of the constructed ceRNA network.

### 3.3 SSRP1 Promotes SMs Proliferation In Vitro

**FIGURE 3:**
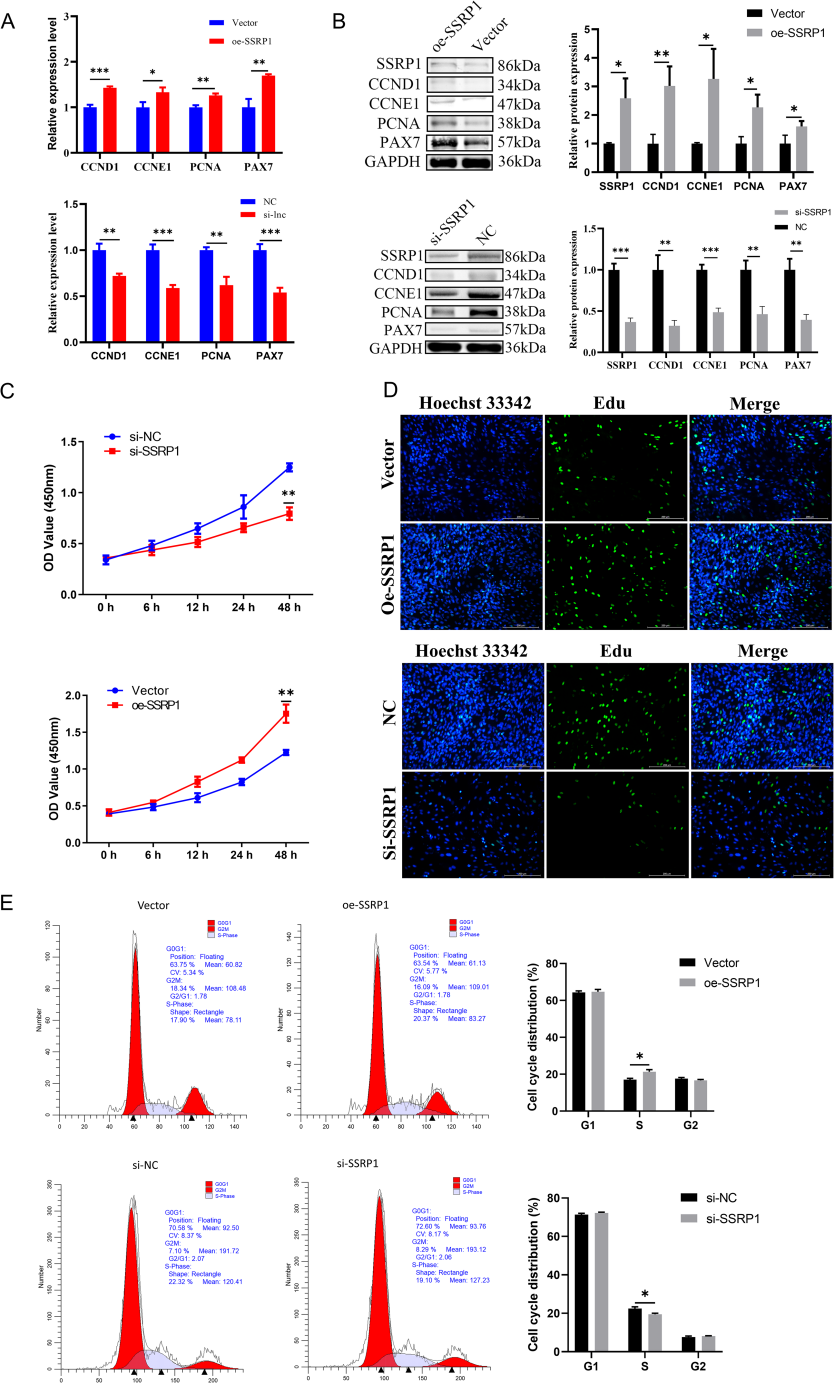
SSRP1 promotes myoblast proliferation in vitro. (A) Relative mRNA levels of CCND1, CCNE1, PCNA and PAX7 were detected in myoblasts transfected with NC, oe‐SSRP1 or si‐SSRP1 using RT‐qPCR. (B) Relative protein levels of CCND1, CCNE1, PCNA and PAX7 were detected in myoblasts transfected with NC, oe‐SSRP1 or si‐SSRP1 using western blot. (C, D) CCK8 assays and EdU assays were performed to determine the ability of proliferation in myoblasts transfected with si‐SSRP1 or NC and oe‐SSRP1 or vector. (E) The cell cycle phase index was analysed by FACS. Data are expressed as the mean ± SEM. *p < 0.05, **p < 0.01, ***p < 0.001.

To investigate the biological function of SSRP1 in SMs, we constructed four siRNAs targeting SSRP1 along with an overexpression vector for SSRP1 (Figure S6A). Among four siRNAs, si‐SSRP1–564 was selected for subsequent experiments because of its high inhibitory efficiency and referred to as si‐SSRP1 (Figure S6B). Overexpression vector oe‐SSRP1 was successfully constructed, resulting in an increased expression level of SSRP1 in SMs (Figure S6C). RT‐qPCR and Western Blot results showed that the mRNA and protein expression levels of cell proliferation markers were significantly increased after SSRP1 overexpression, whereas the opposite was true when SSRP1 was knocked down (Figure 3A,B). CCK8 growth curve analysis showed that increased SSRP1 expression significantly increased cell proliferation capacity, whereas decreased SSRP1 expression had the opposite effect (Figure 3C). Similarly, EdU analysis showed that increased expression of SSRP1 significantly increased the percentage of EdU‐positive cells, but when SSRP1 expression was decreased, the percentage of EdU‐positive cells significantly decreased (Figure 3D). The results of cell cycle studies also showed that overexpression of SSRP1 significantly increased the percentage of cells in S phase (Figure 3E). These results suggest that SSRP1 promotes SMs proliferation.

### 3.4 Overexpression of SSRP1 Facilitate Skeletal Muscle Regeneration In Vivo

**FIGURE 4:**
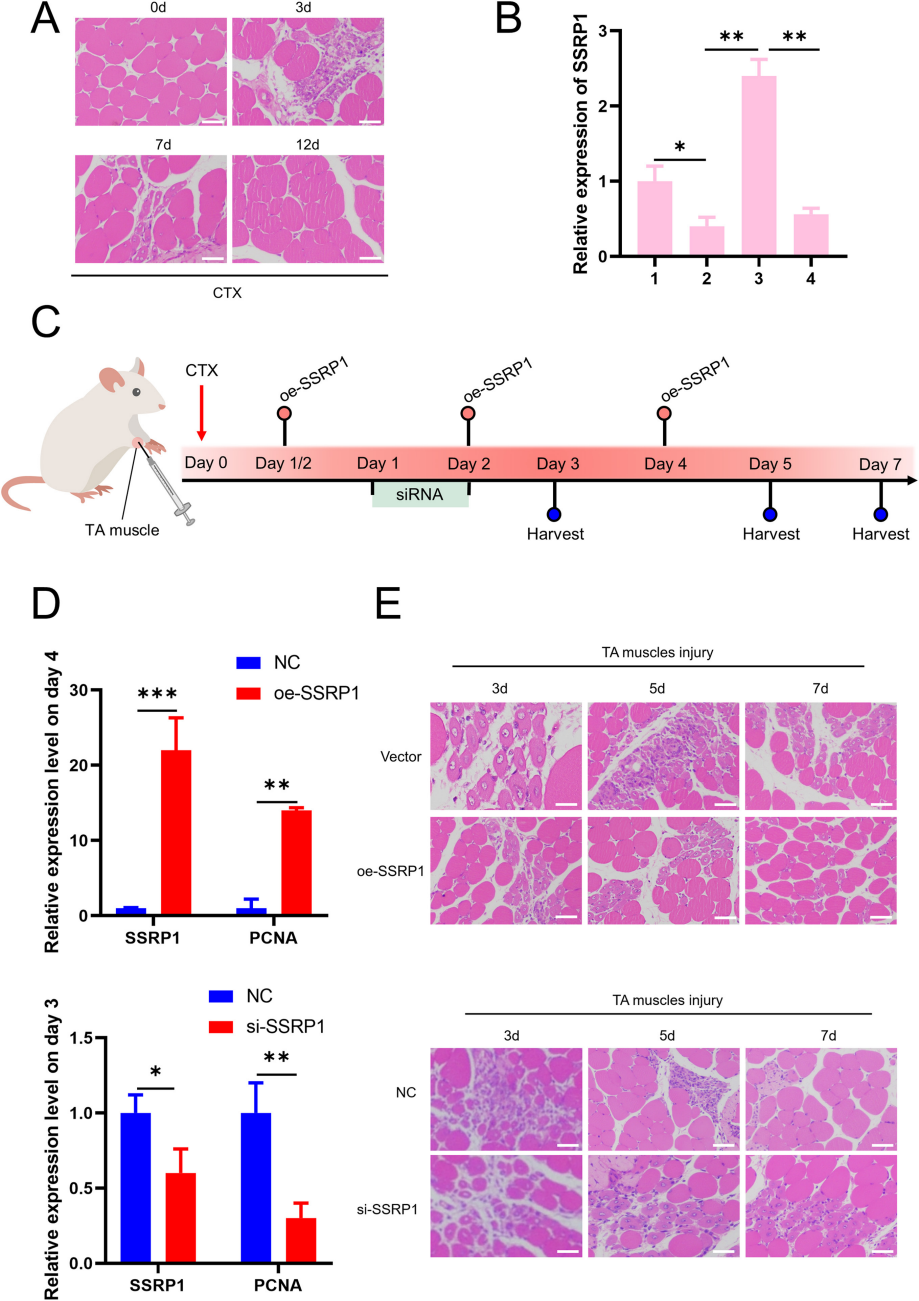
Overexpression of SSRP1 facilitates skeletal muscle regeneration in vivo. (A) HE staining of TA muscles after injection of CTX at 0, 3, 7 and 12 days. (B) The expression of SSRP1 in 1–4 days was detected after CTX treatment by RT‐qPCR. (C) Schematic representation of CTX injury followed by oe‐SSRP1 or si‐SSRP1 treatment in mouse TA muscle. (D) The expression of SSRP1 and PCNA in TA muscle after CTX injury and plasmid or siRNA injection. (E) HE staining was performed on TA muscle 3, 5 and 7 days after transfection of SSRP1 expression plasmid or siRNA in CTX mouse models. Scale bar indicates 50 μm. Data are presented as means ± SEM for three individuals. *p < 0.05, **p < 0.01, ***p < 0.001.

To further verify the function of SSRP1 in skeletal muscle development, we performed homology analysis. The homology of SSRP1 gene between sheep and mouse reached 95% (Figure S7). Therefore, we used the mouse model to further investigate the involvement of SSRP1 in skeletal muscle development in vivo. We established a mouse model of muscle injury. The results of HE staining showed that after CTX treatment, the myofibers of the TA muscle of mice were dissolved into many scattered nuclei (Figure 4A). Significant changes in SSRP1 expression were observed at 1–4 days following CTX treatment (Figure 4B). At Days 1/2, 2 and 4 after CTX treatment, SSRP1 plasmid was transfected to achieve SSRP1 overexpression. Alternatively, siRNA was injected on Days 1 and 2 after CTX treatment to reduce SSRP1 expression (Figure 4C). These resulted in increased SSRP1 and PCNA mRNA levels on Day 4 or decreased levels on Day 3, respectively (Figure 4D). HE staining showed that overexpression of SSRP1 accelerated the rate of muscle repair compared to controls (Figure 4E). Inhibition of SSRP1 expression showed the opposite effect (Figure 4E). Taken together, these results indicate that SSRP1 can promote muscle regeneration, further confirming the involvement of SSRP1 in skeletal muscle development.

### 3.5 Novel‐miR‐400 Suppresses SMs Proliferation In Vitro by Targeting SSRP1

**FIGURE 5:**
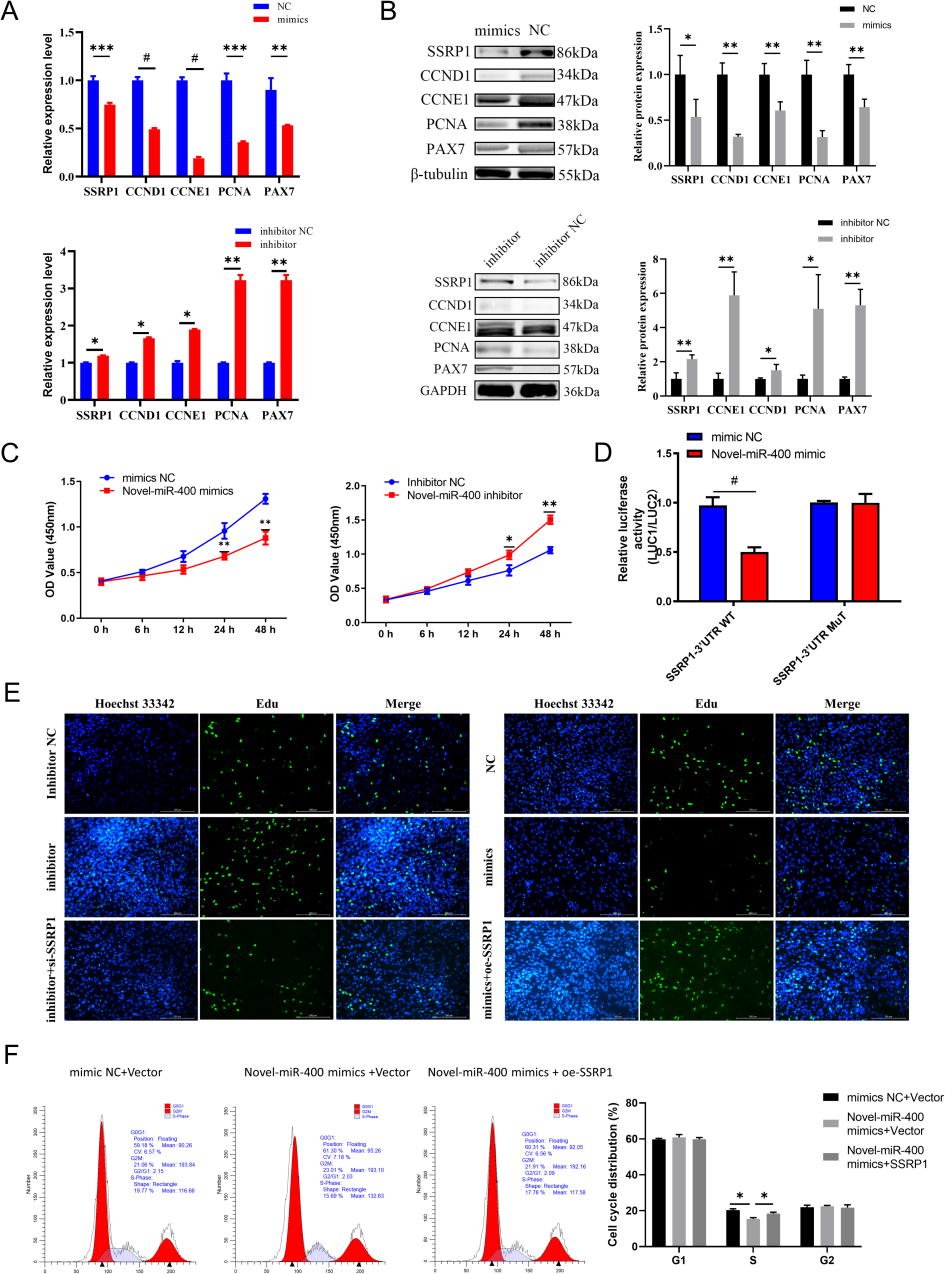
Novel‐miR‐400 suppresses myoblasts proliferation in vitro by targeting SSRP1. (A) Relative mRNA levels of SSRP1, CCND1, CCNE1, PCNA and PAX7 were evaluated by RT‐qPCR in cells transfected with the Novel‐miR‐400 mimics or inhibitor. (B) Relative protein levels of SSRP1, CCND1, CCNE1, PCNA and PAX7 were evaluated by western blot in cells transfected with the Novel‐miR‐400 mimics or inhibitor. (C) CCK8 assays were performed to determine the ability of proliferation in myoblasts transfected with mimics or mimics NC and inhibitor or inhibitor NC. (D) The relative luciferase activities were detected in cells after cotransfection with SSRP1‐WT or SSRP1‐MUT and mimics, inhibitor or NC, respectively. (E) EdU assays were performed to determine the ability of proliferation in myoblasts transfected with indicated mimics, inhibitor, NC, si‐SSRP1 or oe‐SSRP1, respectively. Scale bar, 100 μm. (F) Shift of the GC cell cycle in response to Novel‐miR‐400 mimics was reversed by oe‐SSRP1 as detected by FACS. Data are expressed as the mean ± SEM. *p < 0.05, **p < 0.01, ***p < 0.001, ^#^ *p* < 0.0001.

Given that Novel‐miR‐400 is a newly discovered miRNA, we set out to elucidate its mechanism of action and biological function in SMs. First, after ensuring the successful construction of Novel‐miR‐400 mimics and inhibitors (Figure S8A,B), they were transferred to SMs. The expression of Novel‐miR‐400 was negatively correlated with SSRP1 and cell proliferation markers (Figure 5A,B). The CCK8 assays indicated that increased expression of Novel‐miR‐400 significantly inhibited proliferation viability. Conversely, decreased expression of Novel‐miR‐400 had the opposite effects (Figure 5C). These results suggest that the Novel‐miR‐400 has inhibitory effect on SMs proliferation, and preliminary illustrates SSRP1 could be potential targets of Novel‐miR‐400.

In order to confirm the targeted binding relationship between SSRP1 and Novel‐miR‐400, the online website RNAhybrid was used for binding simulation. The results showed that the minimum free energy (mfe) between SSRP1 and Novel‐miR‐400 was less than −15, and it is highly likely that there is a binding relationship between them (Figure S8C). Then, the luciferase reporter vector GP‐miRGLO was used to clone SSRP1‐WT and SSRP1‐MUT (Figure S8D). These constructs were subsequently cotransfected into SMs along with either Novel‐miR‐400 mimics or NC to evaluate the interaction between Novel‐miR‐400 and SSRP1. The results indicated a significant reduction in the luciferase reporter vector containing the SSRP1 3′UTR‐WT sequence within the Novel‐miR‐400 mimics group. However, these effects were eliminated in the mutant binding site of SSRP1 (Figure 5D). These results indicated that Novel‐miR‐400 could target and bind to the 3′UTR of SSRP1 mRNA and affect its expression.

To further detect whether the effect of Novel‐miR‐400 on SMs was mediated by SSRP1, rescue experiments were designed. Proliferation assay showed that SSRP1 restored the inhibited proliferation of Novel‐miR‐400 mimics (Figure S8E). EdU analysis showed that SSRP1 knockdown reversed the proliferation promotion effect of Novel‐miR‐400 inhibitor in SMs, whereas SSRP1 overexpression counteracted the inhibitory effect caused by Novel‐miR‐400 mimics in SMs (Figure 5E). Meanwhile, cell cycle analysis showed that Novel‐miR‐400 mimics arrested SMs in S phase, whereas overexpression of SSRP1 reversed this effect (Figure 5F). These results suggest that Novel‐miR‐400 inhibits SMs proliferation by targeting the SSRP1 gene.

### 3.6 LOC121818100 Enrichment in the Cytoplasm and Promote Cell Proliferation

**FIGURE 6:**
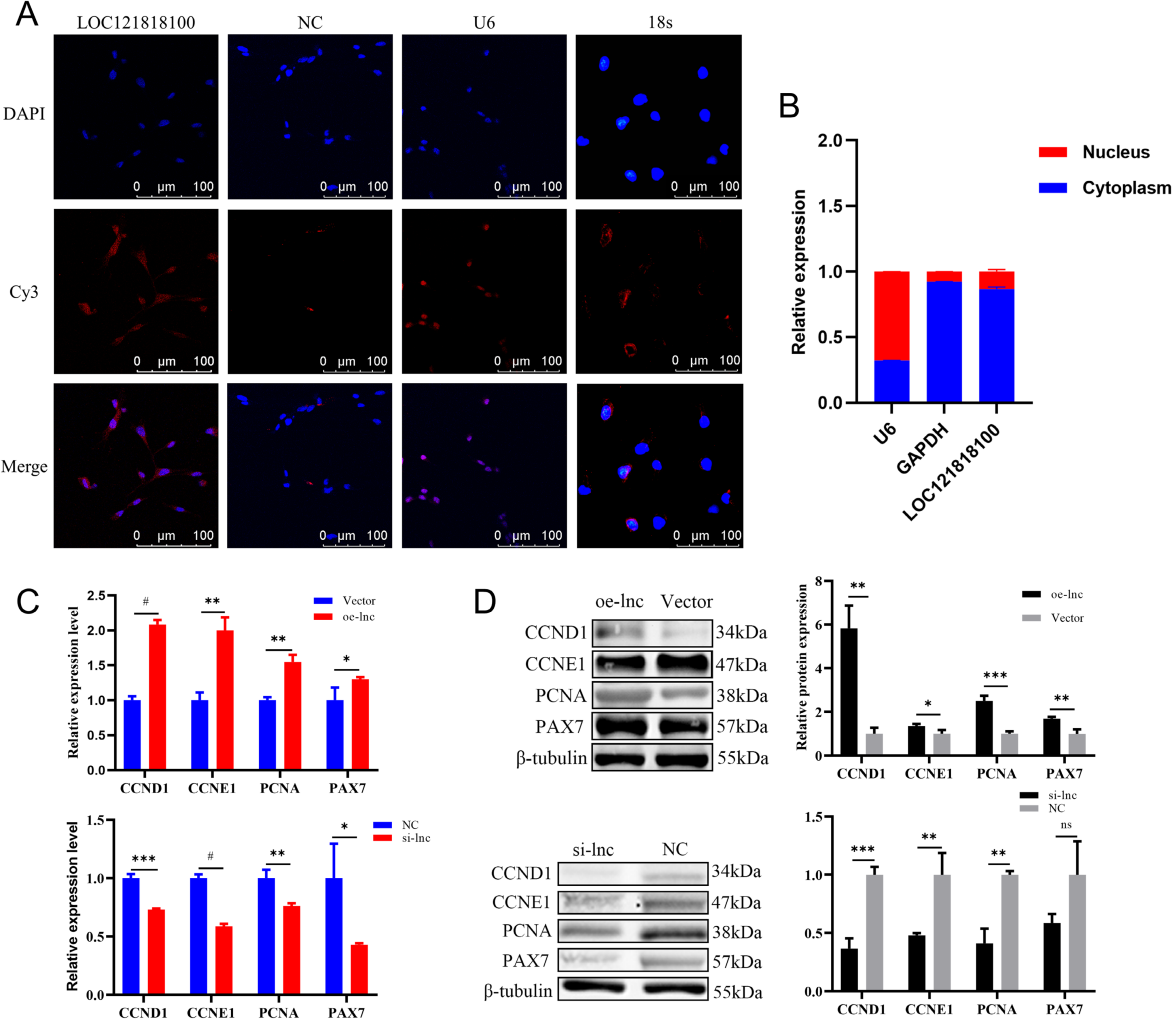
LOC121818100 enrichment in the cytoplasm and promote cell proliferation in vitro. (A) The localization of LOC121818100 detected by FISH. LOC121818100 is labelled by red fluorescence; nuclei are stained by DAPI (blue). Scale bar, 100 μm. (B) LOC121818100 positioning detection by using the nucleus and cytoplasm extraction reagent. (C) Relative mRNA levels of CCND1, CCNE1, PCNA and PAX7 were detected in myoblasts transfected with NC, oe‐lnc and si‐lnc using RT‐qPCR. (D) Relative protein levels of CCND1, CCNE1, PCNA and PAX7 were detected in myoblasts transfected with NC, oe‐lnc and si‐lnc using western blot. Data were showed as mean ± SD; ns indicated no significance. *p < 0.05, **p < 0.01, ***p < 0.001, ^#^ *p* < 0.0001.

Many studies have shown that when lncRNA is located in the cytoplasm, it mainly functions by interacting with miRNA [16]. The subcellular localization of LOC121818100 was observed using FISH and RT‐qPCR (Figure 6A,B). The results show that LOC121818100 mainly exists in the cytoplasm. Thus, the results illustrate that the LOC121818100 has great potential as a sponge for miRNA.

Four siRNAs against LOC121818100 and the overexpression vector of LOC121818100 were constructed (Figure S9A,B). Among four si‐RNAs, si‐lnc–1631 was chosen, renamed si‐lnc, for the following experiment due to its high inhibitory efficiency (Figure S9C). The overexpression vector oe‐lnc of LOC121818100 was successfully constructed, which improved the expression level of LOC121818100 in SMs (Figure S9D). The results of RT‐qPCR and Western Blot showed that expression levels of cell proliferation markers were increased after overexpression of LOC121818100 and decreased after knockdown of LOC121818100 (Figure 6C,D). These results preliminarily suggested that LOC121818100 promotes SMs proliferation.

### 3.7 LOC121818100 Serves as a Sponge of Novel‐miR‐400 to Regulate SSRP1 Expression

**FIGURE 7:**
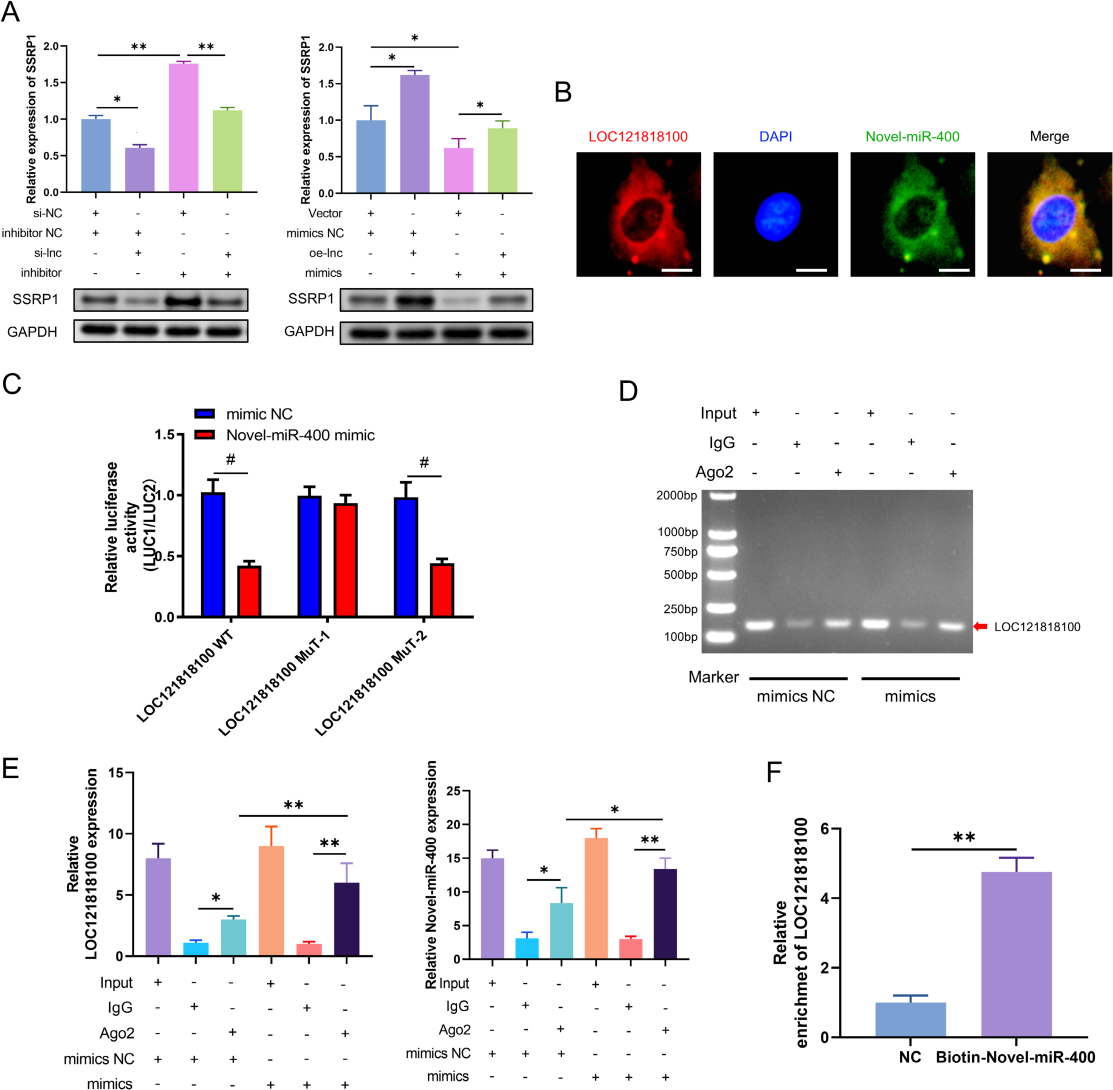
LOC121818100 served as a miRNA sponge of Novel‐miR‐400 to regulate SSRP1 expression. (A) Relative mRNA and protein levels of SSRP1 were determined in cells transfected with the indicated plasmids by RT‐qPCR and western blot, respectively. (B) FISH was performed to observe the cellular location of LOC121818100 (red) and Novel‐miR‐400 (green) in cells (magnification, 400X; scale bar, 10 μm). (C) The relative luciferase activities were detected in cells after cotransfection with LOC121818100‐WT or LOC121818100‐MUT and mimics, inhibitor or NC. (D, E) Anti‐Ago2 RIP assay was executed in myoblasts after transfection with mimics and NC, followed by nucleic acid electrophoresis and RT‐qPCR to detect LOC121818100 and Novel‐miR‐400, respectively. (F) RT‐qPCR analysis of the LOC121818100 level after pulled down biotin‐Novel‐miR‐400 and NC.

lncRNA, as a miRNA sponge, participates in downstream gene regulation, which is an important part of the ceRNA mechanism [17]. To investigate the interaction of LOC121818100, Novel‐miR‐400 and SSRP1 at the cellular level, we conducted a series of experiments. The analysis of SSRP1 expression showed that simultaneously inhibiting or increasing the expression of LOC121818100 and Novel‐miR‐400 would produce antagonistic effects, which would reduce the influence of their respective transfection on SSRP1 expression (Figure 7A).

Considering that lncRNA can act as miRNA sponges in cytoplasm, colocalization FISH assay of lncRNA and miRNA was performed in SMs. In our study, LOC121818100 (red) and Novel‐miR‐400 (green) are both predominantly present in the cytoplasm (Figure 7B). Then, the two most likely binding sites between LOC121818100 and Novel‐miR‐400 were predicted by bioinformatics analysis (Figure S10A). To confirm bioinformatic predictive analysis, we applied dual luciferase reporter gene assays in SMs. The normal full‐length sequence of LOC121818100 (named LOC121818100‐WT) and two mutant sequences that do not bind to Novel‐miR‐400 were subcloned into GP‐miRGLO plasmid (Figure S10B). The results demonstrated a significant decrease in luciferase activity for the LOC121818100‐WT group within the Novel‐miR‐400 mimic group. However, there was no difference in luciferase activity between Novel‐miR‐400 mimic and NC group on LOC121818100‐MuT‐1 group. Moreover, luciferase activity was significantly reduced in the LOC121818100‐MuT‐2 group in the Novel‐miR‐400 mimic group (Figure 7C). These results indicated a direct interaction between LOC121818100 and Novel‐miR‐400, with the true binding site being Site 1 instead of Site 2.

Current studies generally believe that miRNA form RNA‐induced silencing complex (RISC) by binding Argonaute 2 (Ago2), thereby regulating the expression of target genes [18]. Therefore, we performed RIP experiments in SMs to identify RNA molecules that bind to Ago2 proteins. The results of agarose gel electrophoresis and RT‐qPCR showed that anti‐Ago2 could effectively reduce the expression of LOC121818100 and Novel‐miR‐400 compared with the input control. In addition, cells transfected with Novel‐miR‐400 mimics showed a high enrichment of LOC121818100 and Novel‐miR‐400 compared to the NC (Figures 7D,E). RNA pull‐down experiments using biotin‐labelled Novel‐miR‐400 probes further confirmed the direct interaction between LOC121818100 and Novel‐miR‐400 (Figure 7F). Collectively, the data demonstrated that LOC121818100 functions as a sponge for Novel‐miR‐400, thereby promoting SSRP1 expression in SMs.

### 3.8 LOC121818100 Promotes SMs Proliferation Through LOC121818100/Novel‐miR‐400/SSRP1 Axis

**FIGURE 8:**
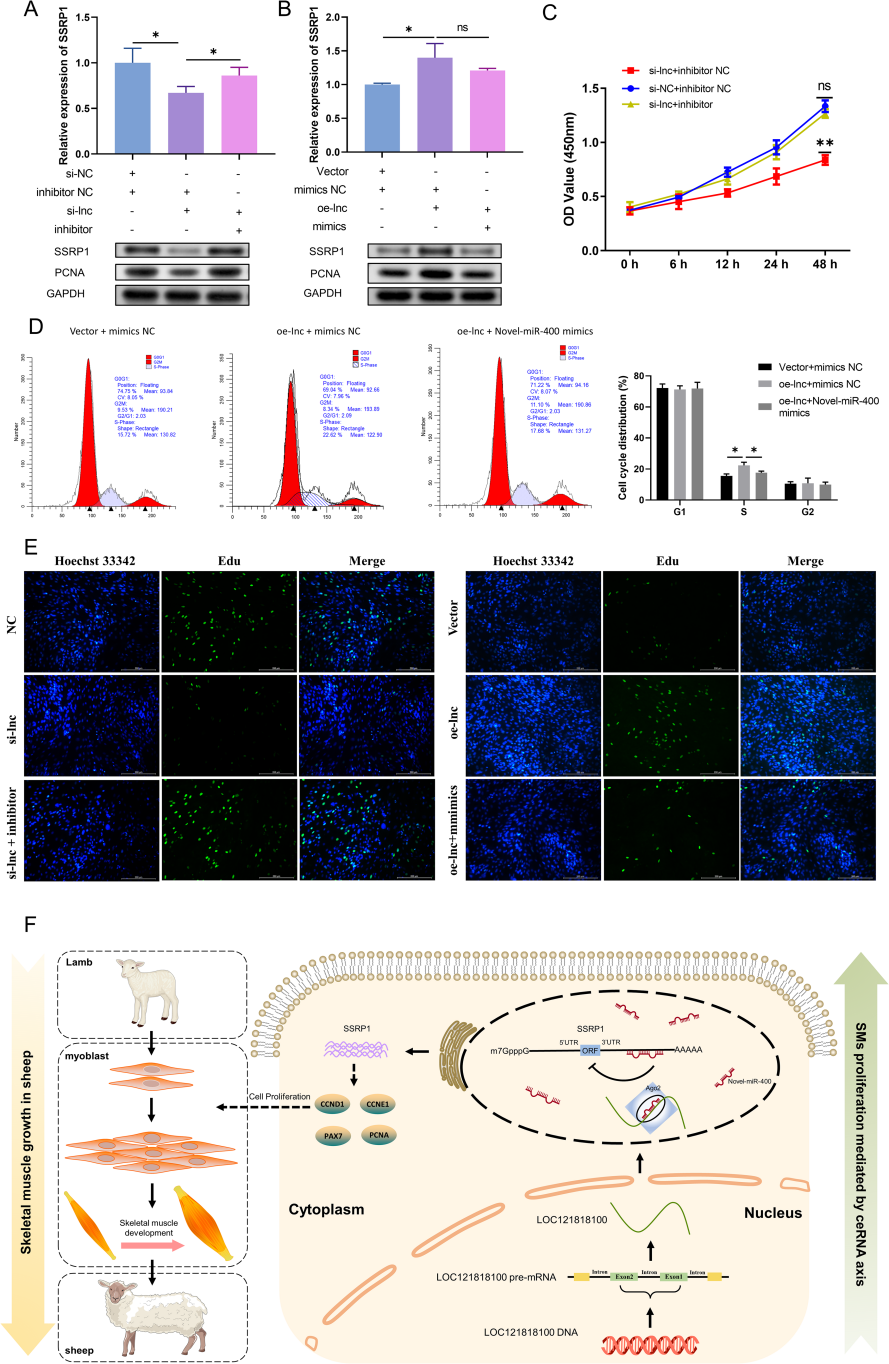
LOC121818100 promotes myoblasts proliferation through LOC121818100/Novel‐miR‐400/SSRP1 axis. (A, B) Relative mRNA and protein levels of SSRP1 and PCNA in cells transfected with mimics, inhibitor, NC, si‐lnc or oe‐lnc by RT‐qPCR and western blot assays. (C) The effects of the si‐lnc on SMs proliferation were antagonized by Novel‐miR‐400 inhibitor. (D) Shift of the cell cycle after transfection of si‐lnc or Novel‐miR‐400 inhibitor combined with si‐lnc detected by FACS. (E) EdU assays were performed to determine the ability of proliferation in cells transfected with mimics, inhibitor, NC, si‐lnc or oe‐lnc. Scale bar, 100 μm. (F) The schematic diagram created with BioGDP.com [19] shows the mechanism underlying LOC121818100 as a ceRNA for Novel‐miR‐400 to regulate SSRP1 expression in the proliferation of SMs and thus affects skeletal muscle growth in sheep. Data were showed as mean ± SD; ns indicated no significance. *p < 0.05, **p < 0.01.

We have confirmed the effects of LOC121818100/Novel‐miR‐400/SSRP1 on SMs. Their interactions at the cellular level were also analysed. However, whether this interaction at the molecular level can actually affect biological function is unknown. Next, we designed rescue experiments using Novel‐miR‐400 inhibitors and mimics. RT‐qPCR and Western blot analyses revealed that the knockdown of LOC121818100 resulted in decreased mRNA and protein levels of SSRP1 and PCNA in SMs (Figure 8A), whereas the result was reversed after upregulation of LOC121818100 expression (Figure 8B). In addition, changes in SSRP1 expression induced by silencing or overexpression of LOC121818100 were reversed by Novel‐miR‐400 inhibitors or mimics, respectively (Figure 8A,B). Finally, the exploration focused on whether Novel‐miR‐400 could also reverse the biological function of LOC121818100 in SMs. The CCK8 assay indicated that the Novel‐miR‐400 inhibitor countered the proliferation inhibition caused by si‐lnc in SMs (Figures 8C). Consistently, Novel‐miR‐400 mimics also counteracted the proliferation‐promoting effects of LOC121818100 overexpression in SMs by flow cytometry and EdU assays (Figure 8D,E). Collectively, the data indicated that LOC121818100 acted as a ceRNA for Novel‐miR‐400, regulating SSRP1 expression and thereby contributing to the progression and development of skeletal muscle in sheep (Figure 8F).

## 4 Discussion

Muscle growth and development are a complex biological process involving multiple cell types and signalling pathways. Previous studies have reported that ceRNA mechanism is involved in muscle growth and injury repair [20]. In this study, we discovered a new ceRNA axis by which we can regulate the SMs proliferation, and the major gene SSRP1 is conserved and has also been shown to promote muscle regeneration in mice. Mechanistic studies have shown that LOC121818100, like a sponge, promotes SMs proliferation by regulating Novel‐miR‐400 inhibition of target gene SSRP1.

As an endogenous ncRNA, lncRNA is involved in various physiological pathways including growth and development [21, 22]. Because most lncRNA contain miRNA response element (MRE), the ceRNA mechanism is the main content of current lncRNA research [23]. Through bioinformatic analysis, we found that LOC121818100/Novel‐miR‐400/SSRP1 axis may play an important role in skeletal muscle development in sheep. Subsequently, LOC121818100 and Novel‐miR‐400 were colocated in the cytoplasm of SMs by a series of experiments including FISH, biotin‐labelled probe pull‐down, dual luciferase reporter gene and RIP. After confirming that LOC121818100 directly binds to Novel‐miR‐400, rescue experiments further confirm that LOC121818100 reverses the proliferation inhibition of Novel‐miR‐400. Our results fully demonstrate the role of LOC121818100 as a sponge for Novel‐miR‐400.

In addition, we found that Novel‐miR‐400 is a key negative regulator of SSRP1. SSRP1 is a multifunctional histone chaperone that binds to Ty homologous 16 inhibitors (SPT16) to form heterodimers that promote the formation of chromatin transcription complex (FATC) [24]. Current research on FACT has focused on SSRP1, one of its components. Existing studies have shown that SSRP1 overexpression is found in various tumour cells compared with normal cells [25]. In addition, in some overproliferating tumour cell lines, compared with the cell lines with low SSRP1 expression, the cell lines with high expression are more prone to cell death, and proliferation inhibition after SSRP1 expression is knocked down [26], suggesting that SSRP1 plays an important role in maintaining cell activity. So far, SSRP1 has been found to affect a variety of physiological activities, including DNA transcription [27], replication [28], cell proliferation [29], differentiation [30] and apoptosis [31]. However, the current research on SSRP1 mainly focuses on the field of tumour, and the research on SSRP1 in other normal tissues is rarely reported. SSRP1 and its upstream regulatory mechanisms involved in skeletal muscle development have not been well defined. Based on previous studies, it can be reasonably speculated that SSRP1 may play an important role in the proliferation and development of myoblasts. Fortunately, through in vitro and in vivo functional studies, we confirm the positive role of SSRP1 in skeletal muscle development and elucidate a novel mechanism by which the LOC121818100/Novel‐miR‐400/SSRP1 axis regulates SMs development.

To our knowledge, this is the first study to delve into the expression, regulation and function of SSRP1 in SMs. These findings will contribute to the breeding and efficient utilization of sheep and may also enlighten the treatment of acute muscle injury and congenital muscle atrophy. However, there are several limitations to the interpretation of our findings. First, the tissues used for RNA‐seq in our study came from a homologous population from a sheep farm, and we cannot rule out that there may be other key ceRNA axes that are also involved in the growth and development of SMs. Second, homologous sequences of LOC121818100 and Novel‐miR‐400 were not found in mice, and our in vitro experiments using the model animal mice only revealed the potential role of SSRP1 in mammalian skeletal muscle growth, whereas validation of the entire regulatory axis needs to be further explored in individual sheep. It should be emphasized that this also provides a new case for the evolution of gene function and reminds us to seriously consider the simplified cognition of ‘whether functional conservation is equivalent to regulatory conservation’. Finally, although the entire ceRNA regulatory axis has been well documented, the downstream regulatory mechanisms of SSRP1 remain to be further explored.

## 5 Conclusion

Our study shows that SSRP1 plays a role in SMs proliferation and acts as a potent proliferating factor to promote skeletal muscle regeneration and development in animals. In sheep, we reveal a potential mechanism by which the LOC121818100/Novel‐miR‐400/SSRP1 axis is involved in skeletal muscle development (Figure 8F). In addition, despite high homology and similar roles, SSRP1 appears to have different regulatory mechanisms in different species. This reveals the evolutionary conservation and regulatory plasticity of gene function to some extent. The discovery will aid breeding efforts and provide new potential targets for treating muscle diseases such as muscular dystrophy. Future studies can further elucidate the decoupling mechanism between function and regulation through multiomics integration and cross‐species gene editing.

## Ethics Statement

This study and all experimental techniques were approved by the Science Research Department (which oversees animal experiments and welfare) of the Institute of Animal Sciences and Chinese Academy of Agricultural Sciences (IAS‐CAAS, Beijing, China). The IAS‐CAAS Animal Ethics Committee (No. IAS2020–82) approved this study. This study was conducted in compliance with the ethical guidelines for authorship and publishing in the Journal of Cachexia, Sarcopenia and Muscle.

## Conflicts of Interest

The authors declare no conflicts of interest.

## Supporting information


**Figure S1.** Full length Western‐blot images.

**Figure S2.** Identification of sheep primary myoblasts. (A) Morphology of sheep primary myoblasts at different periods. Scale bar, 200 μm. (B) Growth curve of sheep myoblast. (C) Immunofluorescence identification of Desmin and MyoD1 of sheep myoblasts. (D) Identification of differentiation ability of myoblasts, Scale bar, 50 μm. DAPI:nuclear staining; FITC:Immunofluorescence; Merge:DAPI and FITC graph overlay.

**Figure S3.** Full length Gel image.

**Figure S4.** Flow Chart of This Study Design.

**Figure S5.** Screening of key lncRNA‐miRNA‐mRNA regulatory axes. (A) GO enrichment analysis of mRNA. (B) mRNA KEGG pathway analysis. (C) Functional items enriched by the top 9 fold change mRNA. (D) UCSC database LOC121818100 expression.

**Figure S6.** Expression efficiency verification of SSRP1 overexpression vector and siRNA. (A) Schematic representation of the specific targeting small interfering RNA (siRNA) and SSRP1 gene structures. (B) RT‐qPCR analysis of SSRP1 mRNA in myoblasts treated with siRNAs. (C) RT‐qPCR analysis of SSRP1 mRNA in myoblasts stably overexpressing SSRP1. Data are expressed as the mean ± SEM. *p < 0.05, **p < 0.01, # *p* < 0.0001.

**Figure S7.** Homology analysis of SSRP1 gene in sheep and mouse at Nucleotide level using NCBI website (https://www.ncbi.nlm.nih.gov/).

**Figure S8.** Expression efficiency verification of Novel‐miR‐400 mimics and inhibitor and targeted binding identification. (A) mimics transfection effectiveness. (B) inhibitor transfection effectiveness. (C) RNAhybrid predicted the binding site for Novel‐miR‐400 in SSRP1–3’UTR. (D) Schematic illustration of SSRP1‐WT and SSRP1‐MUT luciferase reporter vectors. Data are expressed as the mean ± SEM. ***p < 0.001, # *p* < 0.0001.

**Figure S9.** Expression efficiency verification of LOC121818100 overexpression vector and siRNA. (A) and (B) Schematic illustration of the genomic location and structure of sheep LOC121818100 locus and the specific targeting siRNA. (C) RT‐qPCR analysis of LOC121818100 mRNA in myoblasts treated with siRNAs. (D) RT‐qPCR analysis of LOC121818100 mRNA in myoblasts stably overexpressing lOC121818100. Data were showed as mean ± SD. *p < 0.05, **p < 0.01, ***p < 0.001, # *p* < 0.0001.

**Figure S10.** Construction of dual luciferase plasmid of LOC121818100 and targeted binding identification. (A) RNAhybrid predicted the binding site for Novel‐miR‐400 in LOC121818100. (B) Schematic illustration of LOC121818100‐WT and LOC121818100‐MUT luciferase reporter vectors.

**Table S1.** Primer Information in our work.
**Table S2.** The Top 9 up‐ and down‐regulated lncRNA.
**Table S3.** The Top 9 up‐ and down‐regulated mRNA.
**Table S4.** The Top 5 up‐ and down‐regulated miRNA.

**Data S1.** Supplementary Information.
