# Supplementary figures and images for: A Novel ceRNA Axis LOC121818100/Novel‐miR‐400/SSRP1 Regulated Muscle Growth and Injury Repair in Sheep
Source: J Cachexia Sarcopenia Muscle. 2025 Jun 5;16(3):e13836. doi: 10.1002/jcsm.13836 (PMC12138275; doi:10.1002/jcsm.13836)

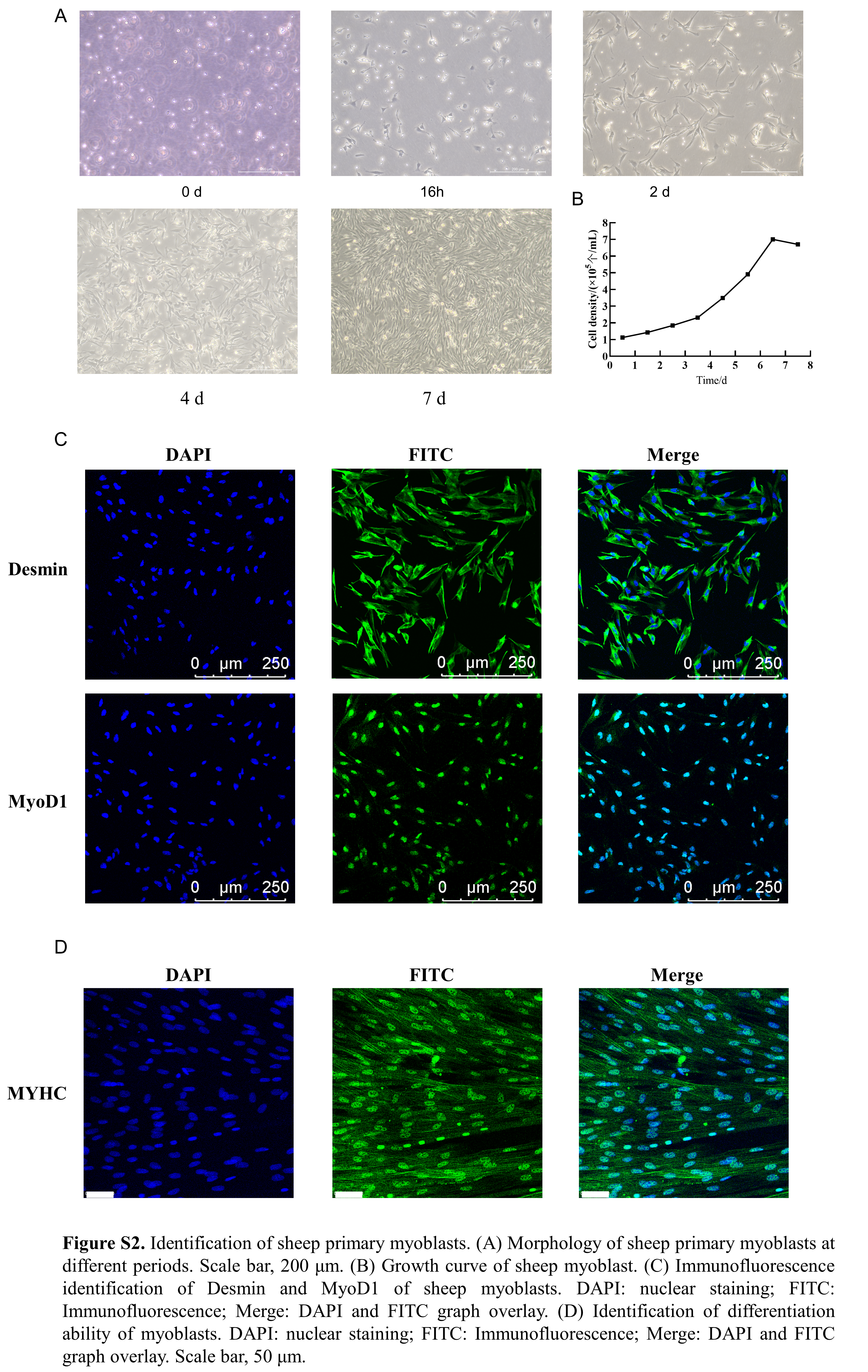

Supplement: Supplementary file 2 — Figure S2. Identification of sheep primary myoblasts. (A) Morphology of sheep primary myoblasts at different periods. Scale bar, 200 μm. (B) Growth curve of sheep myoblast. (C) Immunofluorescence identification of Desmin and MyoD1 of sheep myoblasts. (D) Identification of differentiation ability of myoblasts, Scale bar, 50 μm. DAPI:nuclear staining; FITC:Immunofluorescence; Merge:DAPI and FITC graph overlay. [file JCSM-16-e13836-s003.tif]

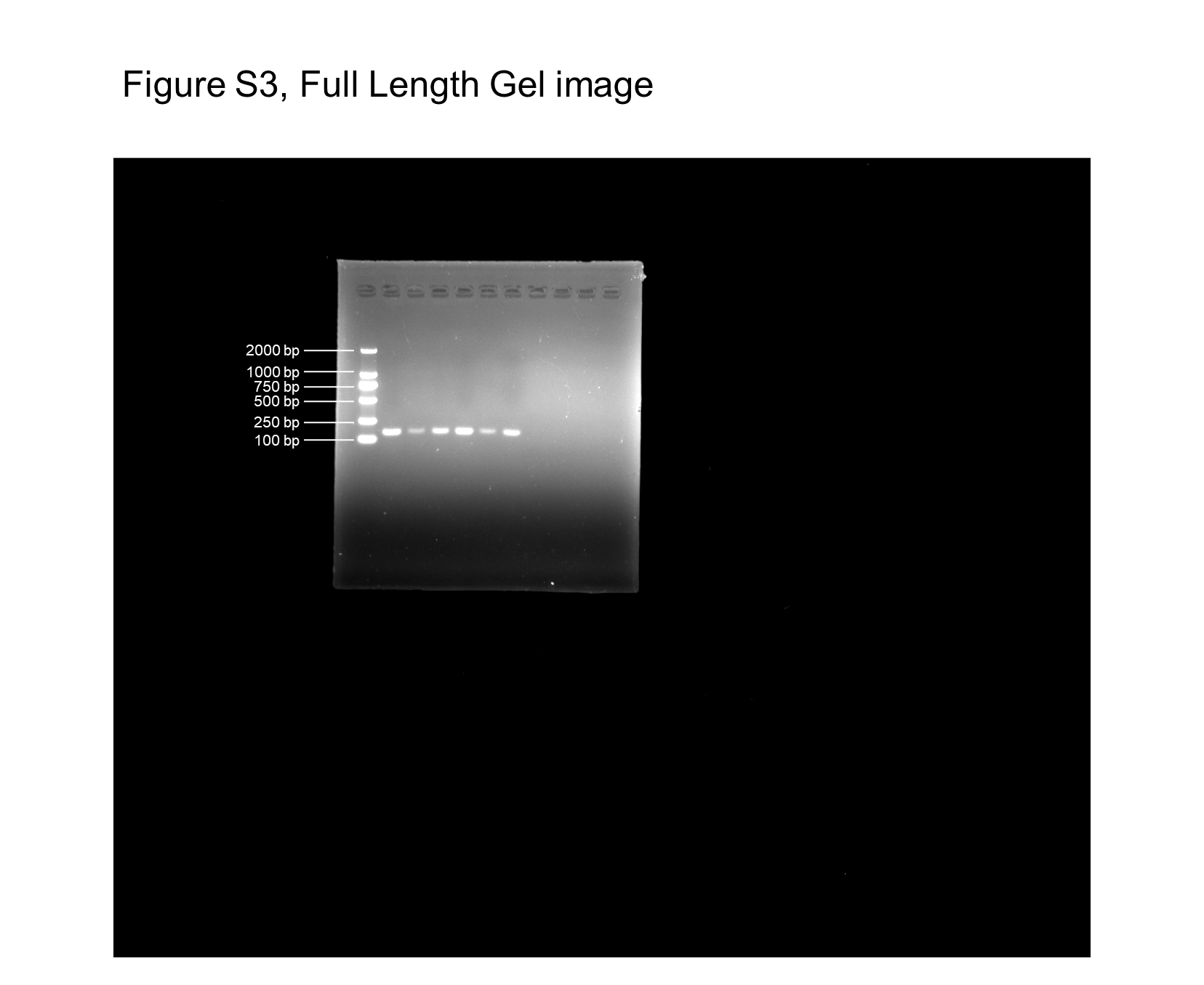

Supplement: Supplementary file 3 — Figure S3. Full length Gel image. [file JCSM-16-e13836-s002.tif]

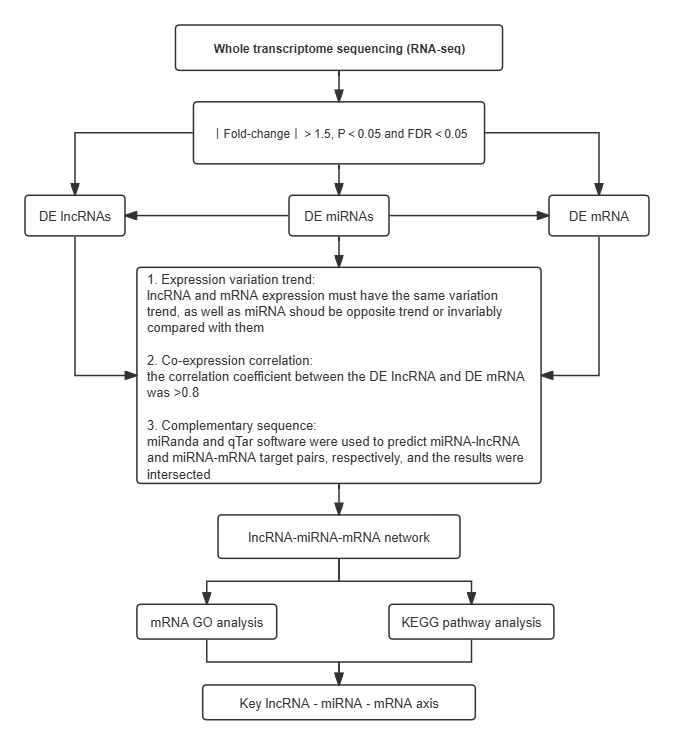

Supplement: Supplementary file 4 — Figure S4. Flow Chart of This Study Design. [file JCSM-16-e13836-s011.png]

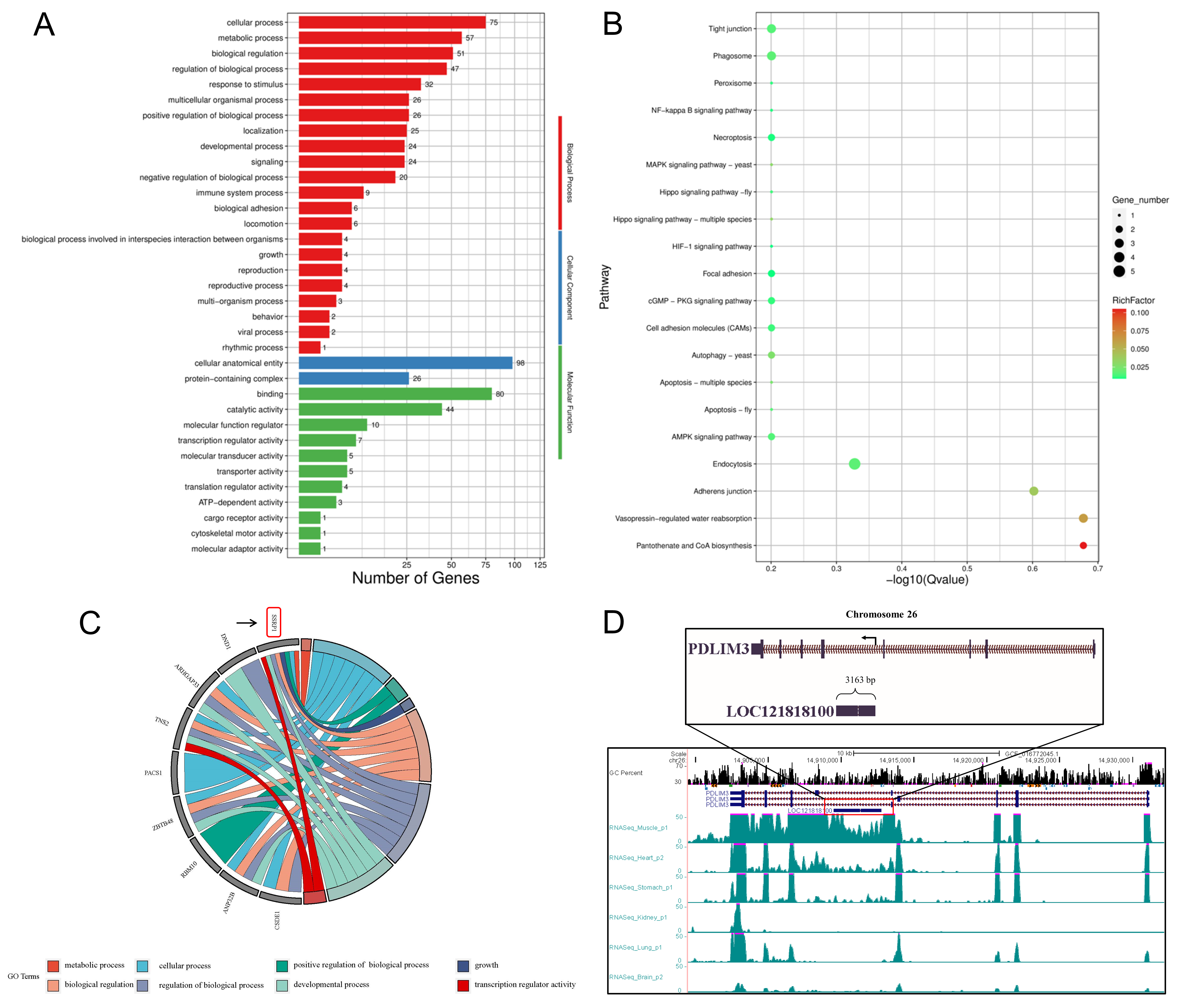

Supplement: Supplementary file 5 — Figure S5. Screening of key lncRNA‐miRNA‐mRNA regulatory axes. (A) GO enrichment analysis of mRNA. (B) mRNA KEGG pathway analysis. (C) Functional items enriched by the top 9 fold change mRNA. (D) UCSC database LOC121818100 expression. [file JCSM-16-e13836-s007.tif]

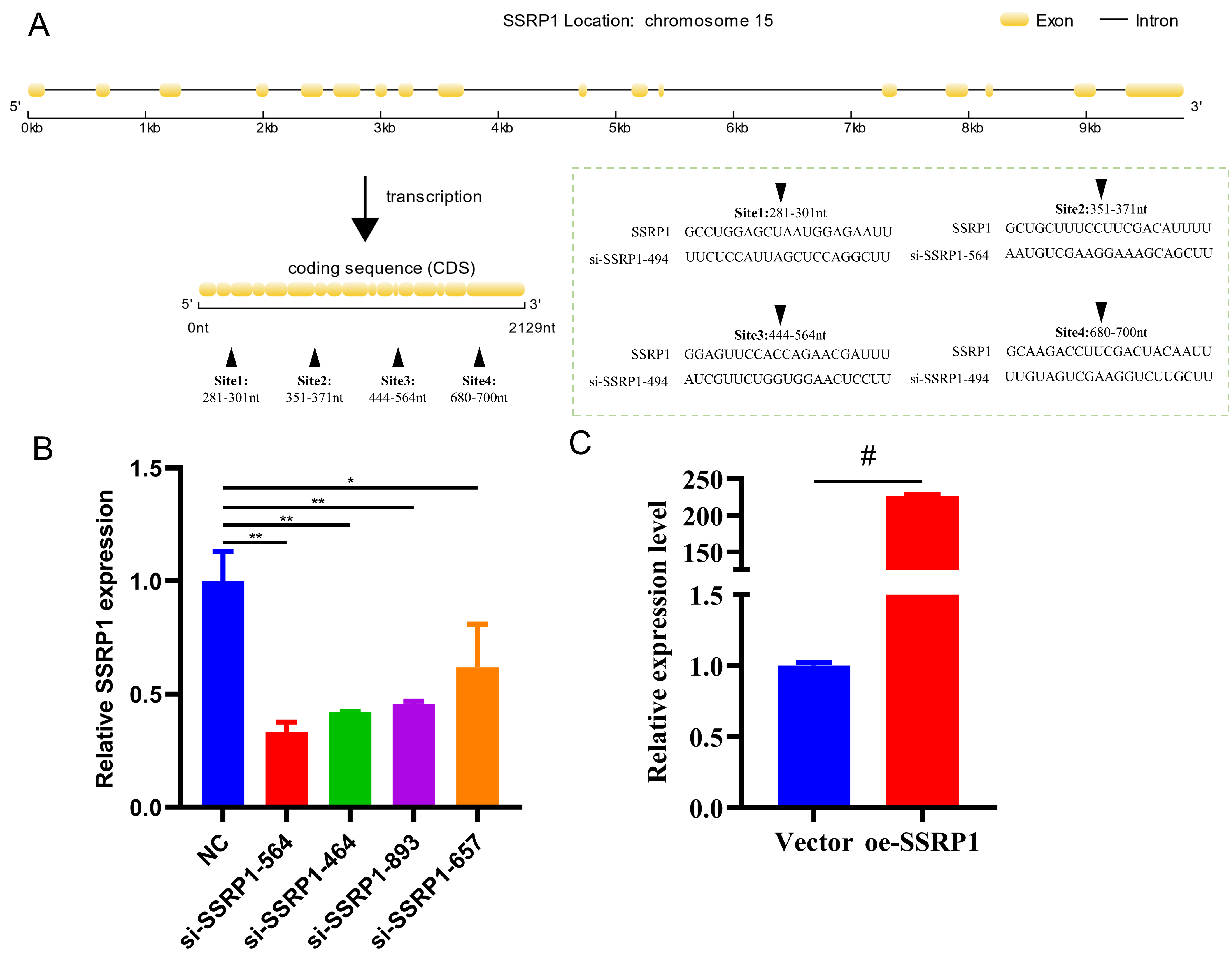

Supplement: Supplementary file 6 — Figure S6. Expression efficiency verification of SSRP1 overexpression vector and siRNA. (A) Schematic representation of the specific targeting small interfering RNA (siRNA) and SSRP1 gene structures. (B) RT‐qPCR analysis of SSRP1 mRNA in myoblasts treated with siRNAs. (C) RT‐qPCR analysis of SSRP1 mRNA in myoblasts stably overexpressing SSRP1. Data are expressed as the mean ± SEM. *p < 0.05, **p < 0.01, # p < 0.0001. [file JCSM-16-e13836-s012.tif]

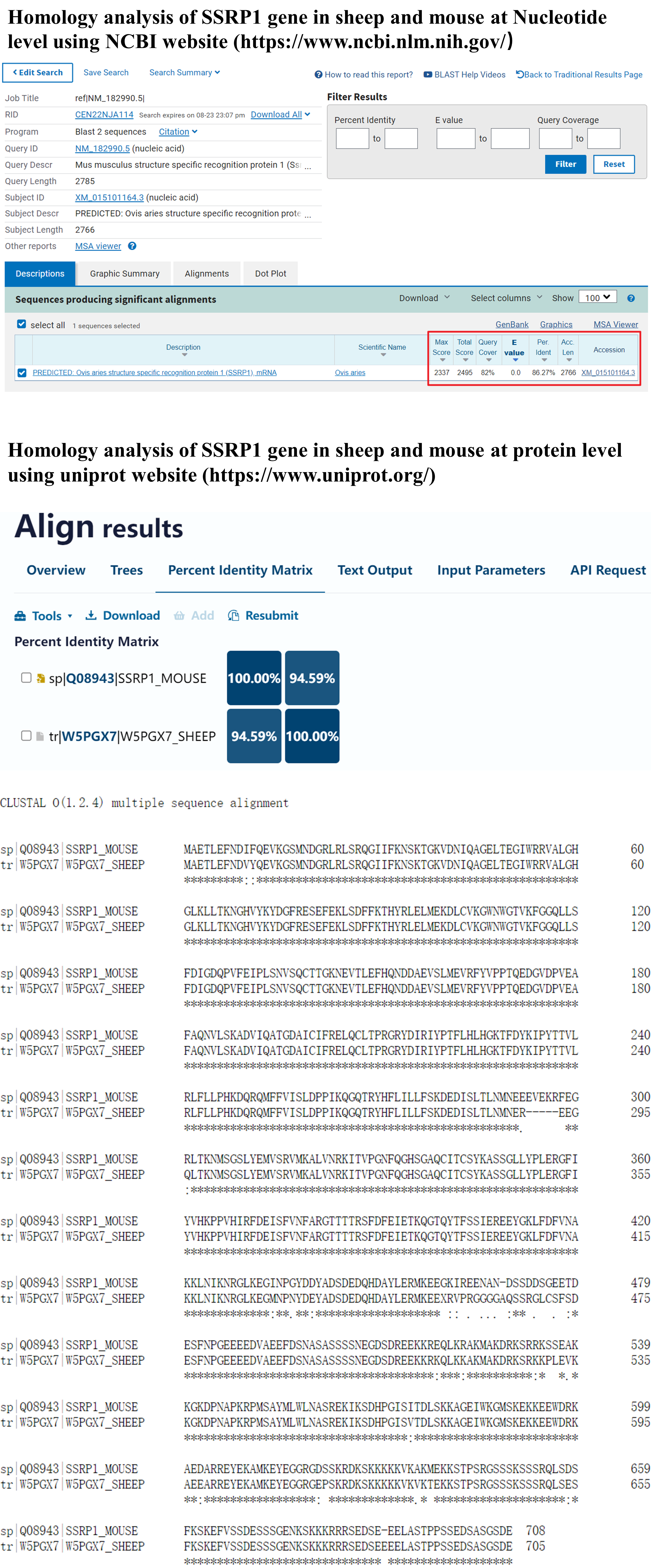

Supplement: Supplementary file 7 — Figure S7. Homology analysis of SSRP1 gene in sheep and mouse at Nucleotide level using NCBI website (https://www.ncbi.nlm.nih.gov/). [file JCSM-16-e13836-s005.tif]

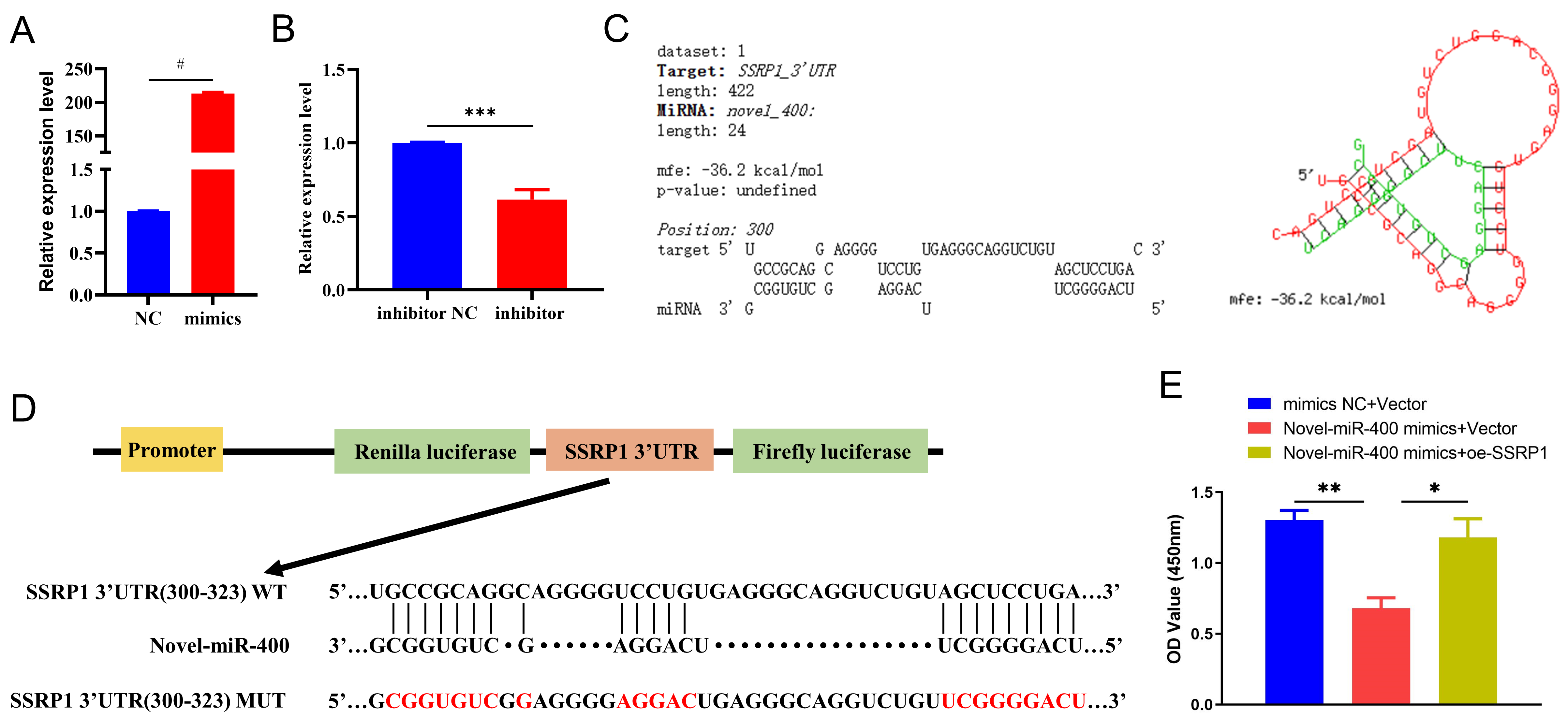

Supplement: Supplementary file 8 — Figure S8. Expression efficiency verification of Novel‐miR‐400 mimics and inhibitor and targeted binding identification. (A) mimics transfection effectiveness. (B) inhibitor transfection effectiveness. (C) RNAhybrid predicted the binding site for Novel‐miR‐400 in SSRP1–3’UTR. (D) Schematic illustration of SSRP1‐WT and SSRP1‐MUT luciferase reporter vectors. Data are expressed as the mean ± SEM. ***p < 0.001, # p < 0.0001. [file JCSM-16-e13836-s006.tif]

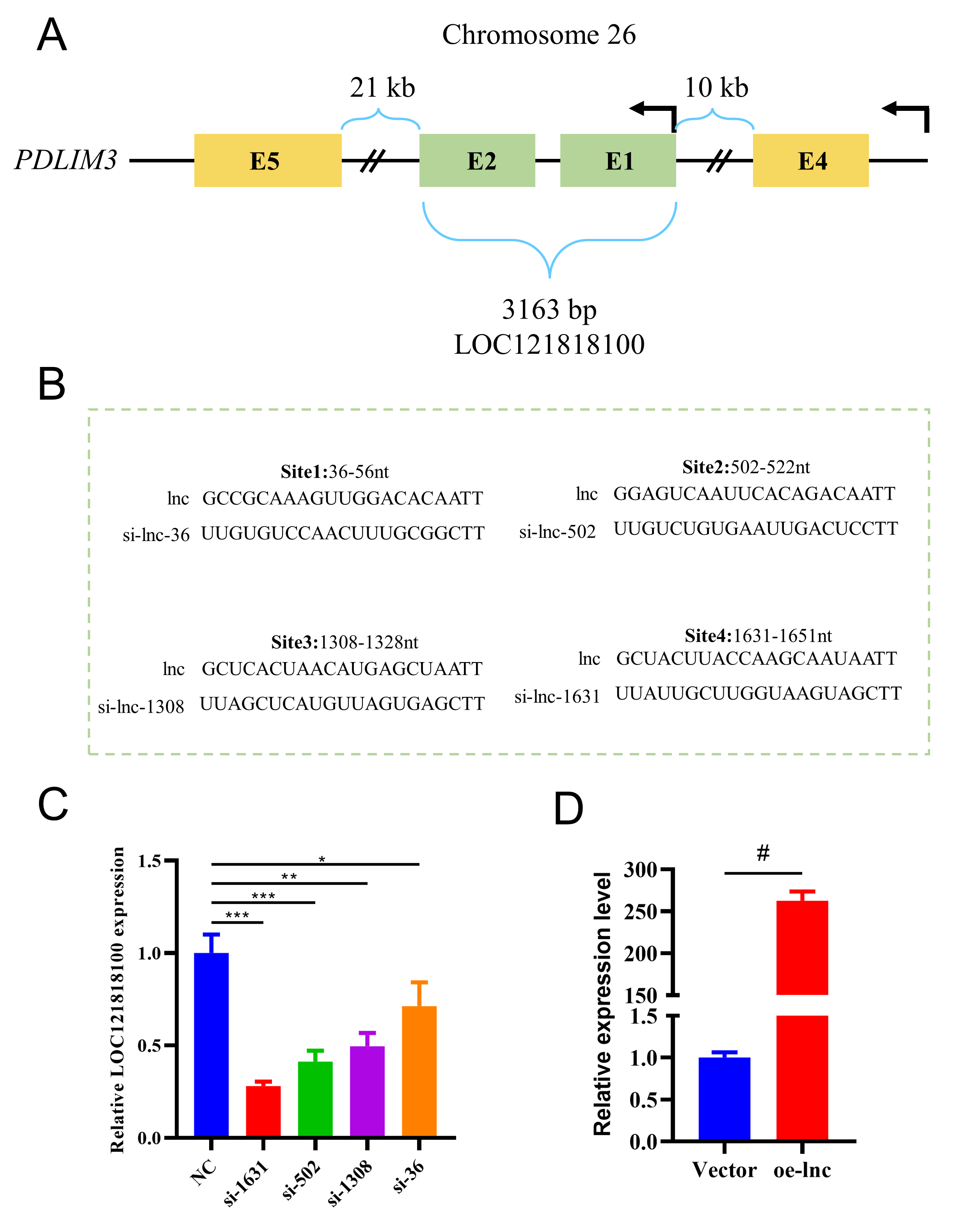

Supplement: Supplementary file 9 — Figure S9. Expression efficiency verification of LOC121818100 overexpression vector and siRNA. (A) and (B) Schematic illustration of the genomic location and structure of sheep LOC121818100 locus and the specific targeting siRNA. (C) RT‐qPCR analysis of LOC121818100 mRNA in myoblasts treated with siRNAs. (D) RT‐qPCR analysis of LOC121818100 mRNA in myoblasts stably overexpressing lOC121818100. Data were showed as mean ± SD. *p < 0.05, **p < 0.01, ***p < 0.001, # p < 0.0001. [file JCSM-16-e13836-s009.tif]

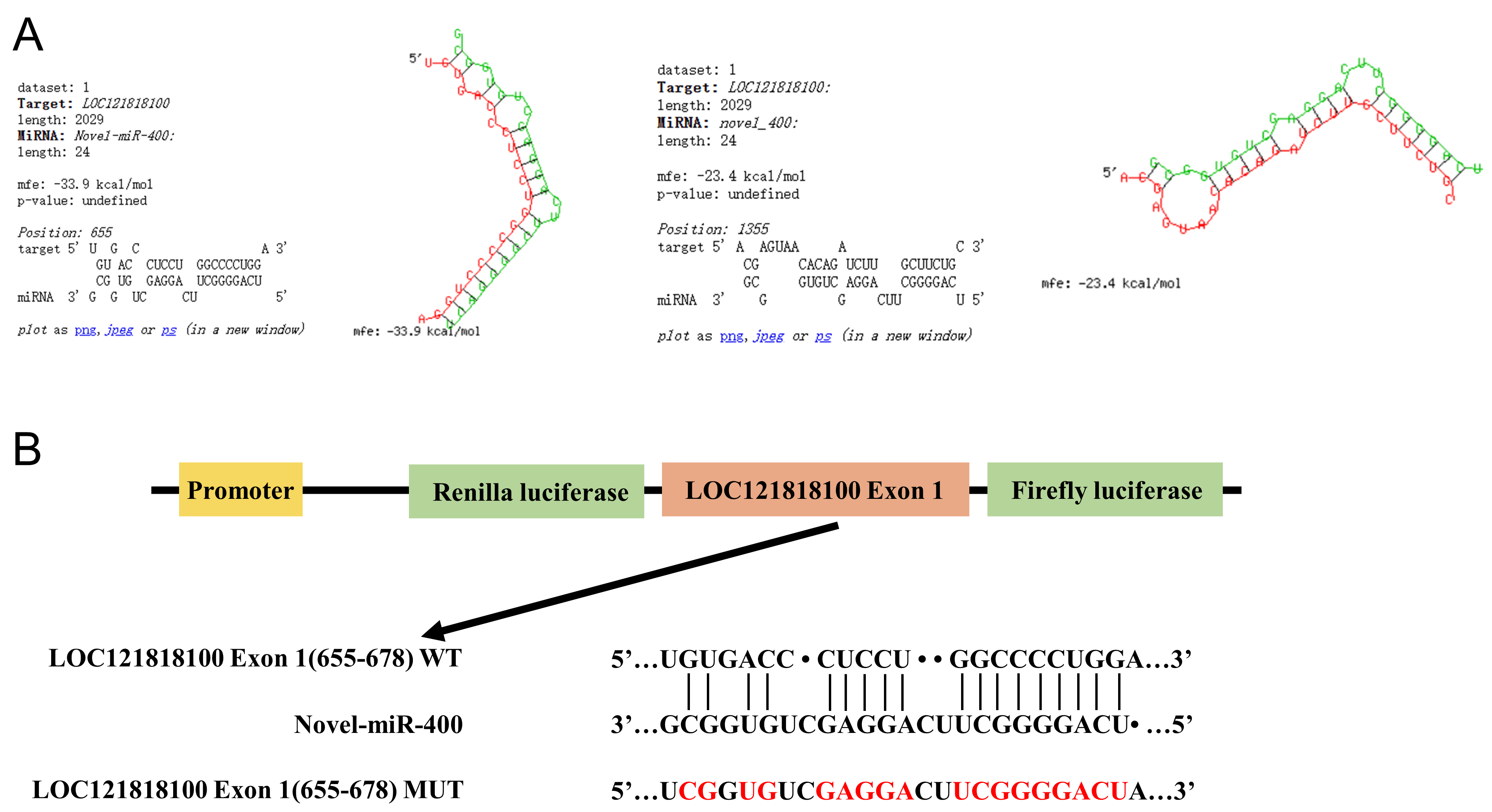

Supplement: Supplementary file 10 — Figure S10. Construction of dual luciferase plasmid of LOC121818100 and targeted binding identification. (A) RNAhybrid predicted the binding site for Novel‐miR‐400 in LOC121818100. (B) Schematic illustration of LOC121818100‐WT and LOC121818100‐MUT luciferase reporter vectors. [file JCSM-16-e13836-s004.tif]
