# Supplementary material for: A Novel ceRNA Axis LOC121818100/Novel‐miR‐400/SSRP1 Regulated Muscle Growth and Injury Repair in Sheep
Source: J Cachexia Sarcopenia Muscle. 2025 Jun 5;16(3):e13836. doi: 10.1002/jcsm.13836 (PMC12138275; doi:10.1002/jcsm.13836)
Supplement: Supplementary file 11 — Table S1. Primer Information in our work. Table S2. The Top 9 up‐ and down‐regulated lncRNA. Table S3. The Top 9 up‐ and down‐regulated mRNA. Table S4. The Top 5 up‐ and down‐regulated miRNA. [file JCSM-16-e13836-s008.docx]

**Table S1.** Primer Information in our work

| Primer name | Primer sequence (5’-3’) |
| --- | --- |
| *GAPDH-F* | CGGCACAGTCAAGGCAGAGAAC |
| *GAPDH-R* | CACGTACTCAGCACCAGCATCAC |
| *SSRP1-F* | CAGACTCGCTACCACTTCCTCATC |
| *SSRP1-R* | CCACCTCCTCCTCGTTCATGTTG |
| *LOC121818100-F* | ACCGAGAGACTAGCACACATACG |
| *LOC121818100-R* | GTAAATCTCCCTCCCTCCCAACTC |
| *Novel-miR-400-F* | GTCAGGGGCTTCAGGAGCTGTGG |
| *U6-F* | GGGCCATGCTAATCTTCTCTGTATCG |
| *CCND1-F* | TTGCTGCTTCCGCCTTGTATC |
| *CCND1-R* | AACCATCCACTTGACACACTTCTC |
| *CCNE1-F* | CGCCCTCGGTGTCCTACTTC |
| *CCNE1-R* | GACCTCCTCCTCGCACTTCTG |
| *PCNA-F* | TTGAAGAAAGTGCTGGAGGC |
| *PCNA-R* | TTGGACATGCTGGTGAGGTT |
| *PAX7-F* | CGTGCCCTCAGTGAGTTCGA |
| *PAX7-R* | CCAGACGGTTCCCTTTGTCG |

**Table S2.** The Top 9 up- and down-regulated lncRNA

| lncRNA_ID | Gene_ID | Regulation | Fold change | P value |
| --- | --- | --- | --- | --- |
| LNC_020844 | XLOC_088325 | up | 30 | 3.64E-23 |
| LNC_020872 | XLOC_088333 | up | 26.30673146 | 3.54E-18 |
| LNC_020857 | XLOC_088327 | up | 24.52144285 | 5.39E-16 |
| LNC_020863 | XLOC_088332 | up | 23.2735568 | 1.47E-14 |
| XR_006055494.1 | LOC121816044 | up | 21.49318332 | 1.27E-12 |
| LNC_003296 | XLOC_014773 | up | 21.41522721 | 1.54E-12 |
| LNC_009316 | XLOC_040982 | up | 21.30707554 | 1.99E-12 |
| LNC_009031 | XLOC_039837 | up | 21.23260524 | 2.38E-12 |
| LNC_004268 | XLOC_018988 | up | 20.86536253 | 5.69E-12 |
| LNC_020870 | XLOC_088333 | down | -25.737392 | 1.83E-17 |
| LNC_018193 | XLOC_077258 | down | -24.13605133 | 1.53E-15 |
| LNC_020869 | XLOC_088333 | down | -23.87358467 | 3.09E-15 |
| LNC_017621 | XLOC_074877 | down | -23.78710934 | 3.86E-15 |
| LNC_004031 | XLOC_017750 | down | -23.00691535 | 2.97E-14 |
| LNC_018209 | XLOC_077288 | down | -22.60523483 | 8.28E-14 |
| XR_006060107.1 | LOC114113097 | down | -22.47174436 | 1.16E-13 |
| LNC_014196 | XLOC_060298 | down | -22.31947481 | 1.70E-13 |
| LNC_013160 | XLOC_056083 | down | -22.28899959 | 1.80E-13 |

**Table S3.** The Top 9 up- and down-regulated mRNA

| Gene | Transcript_ID | Regulation | Fold change | P value |
| --- | --- | --- | --- | --- |
| CUL7 | XM_042237351.1 | up | 29.63557357 | 1.21E-22 |
| VEZF1 | XM_027975376.2 | up | 26.54012744 | 1.79E-18 |
| MACF1 | XM_027968295.2 | up | 26.52203998 | 1.88E-18 |
| LAMA2 | XM_042253450.1 | up | 25.79651394 | 1.54E-17 |
| ZBTB44 | XM_042238009.1 | up | 25.76037111 | 1.71E-17 |
| KALRN | XM_042232379.1 | up | 25.71392108 | 1.95E-17 |
| DST | XM_042237555.1 | up | 25.63784702 | 2.42E-17 |
| OBSCN | XM_042249506.1 | up | 25.00869976 | 1.41E-16 |
| RPS6KA3 | XM_042241830.1 | up | 24.98405331 | 1.51E-16 |
| NEB | XM_042243876.1 | down | -28.87550567 | 1.40E-21 |
| DST | XM_027958742.2 | down | -28.02328542 | 2.04E-20 |
| NR4A3 | XM_042243201.1 | down | -27.83422275 | 3.66E-20 |
| MN1 | XM_042234768.1 | down | -27.79621198 | 4.11E-20 |
| TPM2 | XR_006058632.1 | down | -26.67690803 | 1.19E-18 |
| TNXB | XM_042237090.1 | down | -26.42653954 | 2.49E-18 |
| KCTD20 | XM_027959119.2 | down | -26.26115294 | 4.04E-18 |
| HERC1 | XM_042252348.1 | down | -26.01724763 | 8.18E-18 |
| TAF2 | XM_042254536.1 | down | -25.96337335 | 9.56E-18 |

**Table S4.** The Top 5 up- and down-regulated miRNA

| miRNA_ID | Sequence | Regulation | Fold change | P value |
| --- | --- | --- | --- | --- |
| novel_452 | acguggucgggcucucuuaucucc | up | 4.9 | 0.0036752 |
| novel_502 | guugcucggcggaagugu | up | 3.1304 | 0.022824 |
| novel_345 | aaaggccugaaugaacuuuuuga | up | 2.9736 | 0.038282 |
| novel_227 | uaauuuuugcaaggcuuuuccaa | up | 2.7219 | 0.014917 |
| novel_226 | uaauuuuugcaaggcuuuucca | up | 2.6279 | 0.019467 |
| novel_181 | aucugcucuggcugugaagagg | down | -5.0416 | 0.0022287 |
| novel_363 | cacggcugcccuccggguc | down | -5.0266 | 0.013135 |
| novel_263 | gcuucguacugcuugugagc | down | -4.8786 | 0.015547 |
| novel_259 | uagucucccguugucacuggucu | down | -4.7902 | 0.016621 |
| novel_370 | ucaaacccucuucucccccaga | down | -4.438 | 0.0035205 |

**The sequence of Novel miR-400, the object of this study, is as follows:**

ucaggggcuucaggagcuguggcg
