# Supplementary material for: A Novel ceRNA Axis LOC121818100/Novel‐miR‐400/SSRP1 Regulated Muscle Growth and Injury Repair in Sheep
Source: J Cachexia Sarcopenia Muscle. 2025 Jun 5;16(3):e13836. doi: 10.1002/jcsm.13836 (PMC12138275; doi:10.1002/jcsm.13836)
Supplement: Supplementary file 12 — Data S1. Supplementary Information. [file JCSM-16-e13836-s001.pdf]

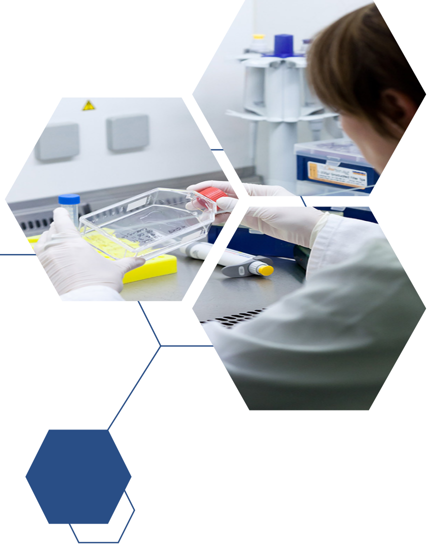

in vivo-jetPEI®

in vivo DNA & siRNA/miRNA transfection reagent

PROTOCOL

DESCRIPTION

***in vivo*-jetPEI®** is a linear polyethylenimine, which mediates efficient nucleic acid (DNA, shRNA, siRNA, miRNA, oligonucleotides, …) delivery to a wide range of tissues using various delivery routes: intravenous (IV), intraperitoneal (IP), intratumoral, subcutaneous, topical, intrathecal, etc. Upon IV administration, high levels of nucleic acid delivery are achieved into the lungs. Other organs such as salivary glands, heart, spleen and liver are also targeted following IV injection. In addition, ***in vivo*-jetPEI®** is an effective carrier for local gene and siRNA delivery such as intratumoral or topical application on the skin.

Previous publications using ***in vivo*-jetPEI®** can be found in the Polyplus-transfection database, available online at www.polyplus-transfection.com/resources/cell-transfection-database/.

1 *in vivo* Transfection Protocol 3
1.1 Reagents Required 3
1.2 Recommended Amount of Nucleic Acid and Injection Volume 3
1.3 Protocol 5
2 Troubleshooting 8
3 Product Information 9
3.1 Ordering Information 9
3.2 Content 9
3.3 Reagent Use and Limitations 9
3.4 Quality Control 9
3.5 Formulation and Storage 9
3.6 Definition of N/P ratio 9
3.7 Trademarks 10
3.8 Contact Information 10

1 *IN VIVO* TRANSFECTION PROTOCOL

1.1 REAGENTS REQUIRED

We recommend using the 10% isotonic glucose solution (w/v) provided in the kit. This is required in order to form small and stable nucleic acid/*in vivo*-jetPEI® complexes. The use of ionic buffers such as PBS or cell culture media for complex preparation should be avoided.

The nucleic acid should be resuspended in low salt buffer since high salt content in the nucleic acid preparation may lead to precipitation upon complexes formation.

For DNA, the best results are achieved with high quality endotoxin free DNA resuspended in ddH_2O and a stock solution of 3-7 µg/µL.

For si/miRNA, it is preferable to use high quality grade si/miRNA (PAGE or HPLC purification) and a stock concentration of 5-10 µg/µL.

1.2 RECOMMENDED AMOUNT OF NUCLEIC ACID AND INJECTION VOLUME

The amount of nucleic acid to deliver should be determined according to the animal model, the administration route and the targeted organ. Recommendations for delivery of DNA, siRNA, oligonucleotides and shRNA-expressing plasmids in rodents are given in Table 1.

The concentration of nucleic acid in the final injection solution should not exceed **0.5 µg/µL**.

The volume of reagent is defined by the N/P ratio and is calculated according to the formula on page 8. As a general guideline, we recommend using: **N/P** = **6 – 8**. (*i.e.* 0.12 to 0.16 µL of *in vivo*-jetPEI® per µg of nucleic acid). Prior to injections, ensure that *in vivo*-jetPEI® and glucose solution are equilibrated at room temperature.

Table 1. Recommended conditions for most common injection routes in mice and rats

Animal	Site of injection	Starting conditions	Nucleic acid optimization range	Injection volume optimization range (5% glucose)
Mouse	IV<br>Tail vein/retro-orbital	40 µg nucleic acid<br>6.4 µL reagent<br>200 µL of 5% glucose	40 - 60 µg	200 - 400 µL
	IP	100 µg nucleic acid<br>16 µL reagent<br>500 µL of 5% glucose	100 - 200 µg	400 - 600 µL
	Intratumoral	10 µg nucleic acid<br>1.2 µL reagent<br>50 µL of 5% glucose	5 - 15 µg	20 - 100 µL
	Subcutaneous (s.c)	20 µg nucleic acid<br>3.2 µL reagent<br>100 µL of 5% glucose	20 - 30 µg	100 – 200 µL
	Intracerebral	1.5 µg nucleic acid<br>0.12 µL reagent<br>3 µL of 5% glucose	1 - 2 µg	2 - 4 µL
	Intradermal	5 µg nucleic acid<br>0.6 µL reagent<br>20 µL of 5% glucose	5 - 10 µg	20 – 50 µL
Rat	IV	150 µg nucleic acid<br>24 µL reagent<br>1 mL of 5% glucose	100 - 300 µg	1 - 1.5 mL
	Intracerebral	3 µg nucleic acid<br>0.36 µL reagent<br>10 µL of 5% glucose	2 - 4 µg	8 - 10 µL

Depending on the application, multiple injections may be required and we recommend to keep the frequency of injection to every 2 – 3 days, with a maximum of 3 injections per week per animal.

For other administration routes (*e.g.* Fig. 1) such as intravitreal, nasal instillation, intra-arterial, intradermal, intracortical (kidney), bladder instillation, intratesticular etc., please contact our technical support at support@polyplus-transfection.com for advice or browse the literature on our website http://www.polyplus-transfection.com/resources/cell-transfection-database/

Figure 1. Successful delivery routes in mouse

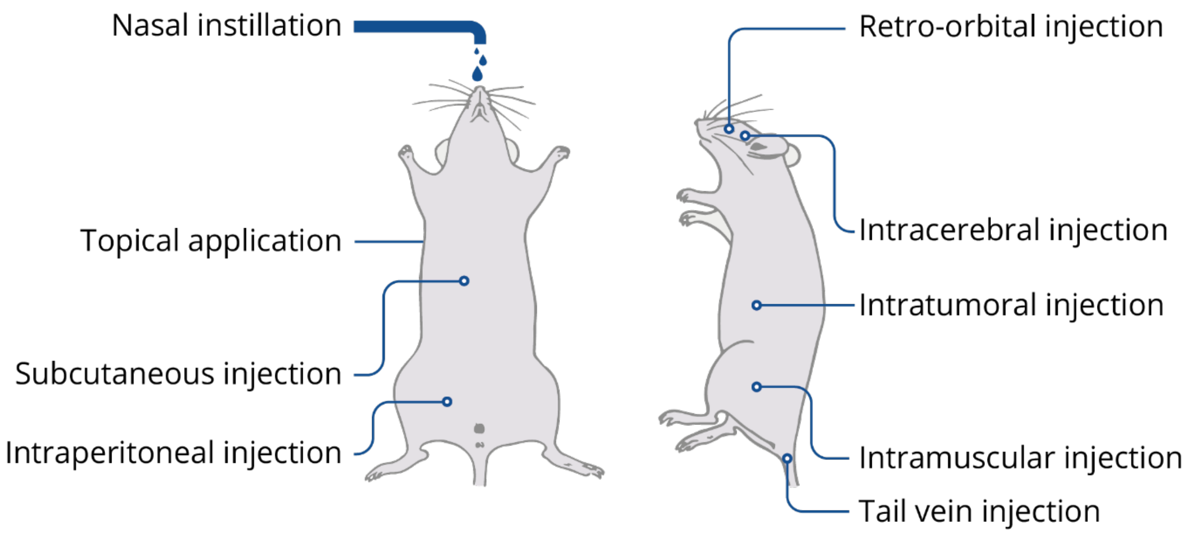

Experimental guidelines for other animal models **such as chicken, quail, sheep, dog, monkey etc.** are available **from our *in vivo* specialists.** You will be amazed by the wide range of animal models we have developed protocols for.

1.3 PROTOCOL

The preparation of the *in vivo*-jetPEI®/nucleic acid complexes should be performed in a laminar flow hood using a 10% glucose solution (provided with reference number 201-10G and 201-50G). The final concentration of glucose in the injection volume should be 5%.

We recommend preparing a mastermix to ensure homogenous complex formation, the smallest mix being minimum 50 µL.

Define the experimental protocol and parameters:

- Set the injection volume of complexes to be prepared per animal (Table 1).
  Note: the final concentration of glucose in the injection volume is 5%.
- Define the amount of nucleic acid to be delivered per injection (Table 1).
  Note: the final concentration of nucleic acid in the injection volume should not exceed 0.5 µg/µL.
- Choose the N/P ratio. As a general guideline, we recommend using: **N/P = 6 – 8 (*i.e.* 0.12 to 0.16 µL of *in vivo*-jetPEI® per µg of nucleic acid).**
- Calculate the corresponding volume of *in vivo*-jetPEI® (Table 2).
  Note: use lower amounts of nucleic acid when using high N/P ratios.

Table 2. Volumes of *in vivo*-jetPEI® to be used according to the N/P ratio and the amount of nucleic acid required

Amount of nucleic acid (µg)	Volume (µL) of *in vivo*-jetPEI®	
	N/P = 6	N/P = 8
1	0.12	0.16
5	0.6	0.8
10	1.2	1.6
40	4.8	6.4
50	6	8
100	12	16

Protocol overview

For homogeneous complex preparation, the nucleic acid solution should represent one half of the injection volume and the *in vivo*-jetPEI® reagent solution should represent the other half of the injection volume.

1. Dilute the nucleic acid into ½ the injection volume in 5% glucose (final concentration) using the 10% glucose stock solution (provided) and sterile water. Vortex gently or mix by pipetting up and down.
2. Vortex *in vivo*-jetPEI® reagent for 5 sec and spin down before use.
3. Dilute the *in vivo*-jetPEI® reagent into ½ the injection volume in 5% glucose (final concentration) using the 10% glucose stock solution (provided) and sterile water. Vortex gently and spin down.
4. Add the diluted *in vivo*-jetPEI® to the diluted nucleic acid all at once, vortex gently and spin down.
5. Incubate for 15 minutes at room temperature. From this time point, the complexes are stable 4 h at room temperature and for up to 7 days when stored at 4 °C.
6. Perform injections into animals using complexes equilibrated at room temperature.
   If required, injections can be repeated up to 3 times a week.
7. Monitor gene expression as required at the appropriate time point (6 – 72 h after the last injection) depending on the mode of injection and the targeted organ.

__Example__*: *IV injection in mouse

Preparation of 200 µL injection volume of 5% glucose containing 40 µg of plasmid DNA and in vivo-jetPEI® at N/P = 8

1. Dilute 40 µg of DNA into 50 µL of 10% glucose; add sterile water to 100 µL, vortex gently and spin down.
2. Dilute 6.4 µL of *in vivo*-jetPEI® into 50 µL of 10% glucose; add sterile water to 100 µL, vortex gently and spin down.
3. Add the diluted *in vivo*-jetPEI® to the diluted DNA at once, vortex briefly and spin down.
4. Incubate for 15 minutes at room temperature.
5. Perform injections into animals using complexes equilibrated at room temperature.
6. Monitor gene expression.

Protocol for nucleic acid/*in vivo*-jetPEI® complexes preparation

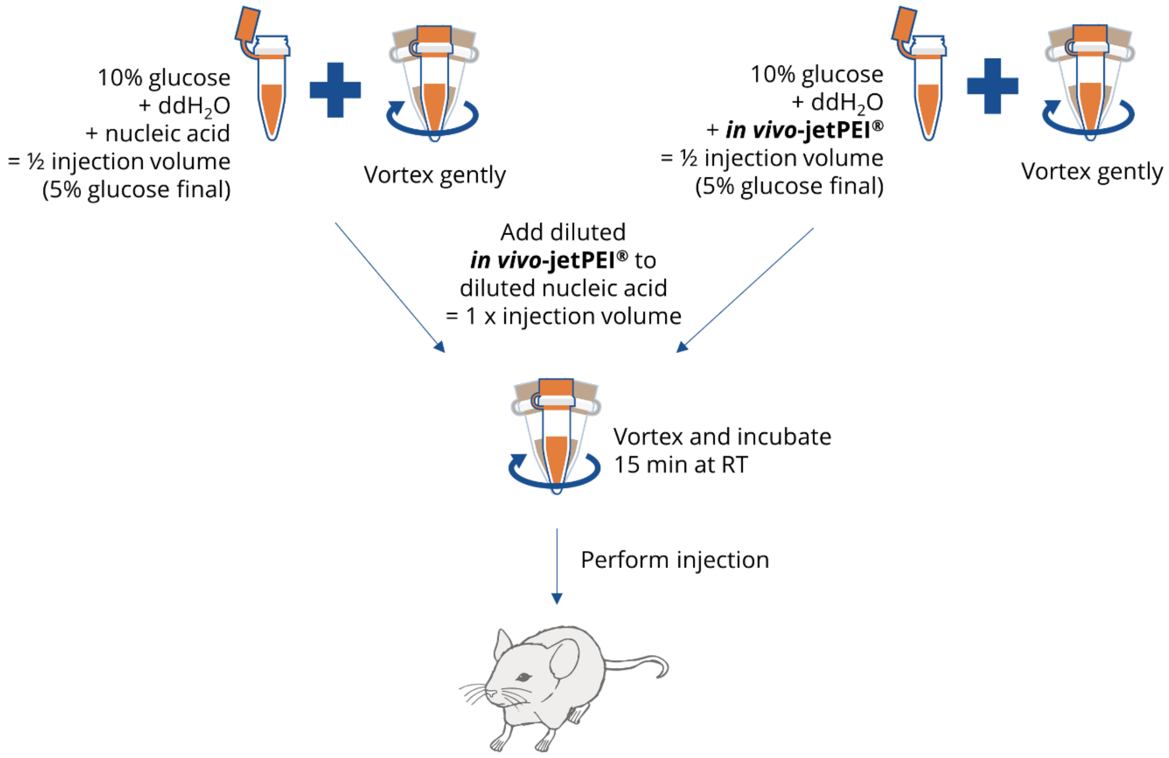

2 TROUBLESHOOTING

Observations	Actions
**Unsatisfactory results**	• Optimize the amount of nucleic acid used in the delivery assay.<br>• Optimize the injection volume.<br>• Use high quality plasmid or si/miRNA preparation. Ensure they contain neither salt, RNA, protein nor endotoxin. For plasmid DNA, $OD_{260/280}$ ratio should be greater than 1.8. It is best to use DNA prepared in water. For si/miRNA, prefer HPLC or PAGE purified oligos.<br>• Optimize the N/P ratio.<br>• Check that the nucleic acid is efficient *in vitro*.<br>• Ensure that the complexes are prepared in glucose 5%.<br>• Ensure that both nucleic acid and *in vivo*-jetPEI® are diluted in 5% glucose before mixing.
**Toxicity**	• Decrease the amount of nucleic acid, keeping the N/P ratio constant.<br>• Decrease the N/P ratio, keeping the amount of nucleic acid constant.<br>• If using plasmid DNA, ensure the preparation is endotoxin-free and DNA is resuspended in water.<br>• Ensure that the N/P ratio is lower than 8 (0.16 µL *in vivo*-jetPEI® per µg of DNA).

3 PRODUCT INFORMATION

3.1 ORDERING INFORMATION

Ref. N°	*in vivo*-jetPEI® Reagent	10% Glucose solution, sterile filtered 0.2 µm
**201-10G**	0.1 mL	10 mL
**201-50G**	0.5 mL	2 x 10 mL

3.2 CONTENT

100 µL of *in vivo*-jetPEI® is sufficient to perform 15-25 intravenous injections in mouse. A 10% glucose solution is included to prepare the *in vivo*-jetPEI®/nucleic acid complexes. This solution should be used to ensure successful delivery experiments.

3.3 REAGENT USE AND LIMITATIONS

For research use only. Not for use in humans.

3.4 QUALITY CONTROL

Each batch of *in vivo*-jetPEI® reagent is tested for conformity to established Quality Controls and relevant specifications. A Certificate of Analysis is provided with each vial of reagent.

3.5 FORMULATION AND STORAGE

in vivo-jetPEI® is provided at 150 mM (expressed as the concentration of nitrogen residues) in sterile apyrogenic water. *in vivo*-jetPEI® and 10% glucose should be stored at -20 °C upon arrival for long term storage. When stored appropriately, *in vivo*-jetPEI® is stable at least one year, as guaranteed and indicated on the Certificate of Analysis.

Polyplus-transfection® has been awarded ISO 9001 Quality Management System Certification since 2002, which ensures that the company has established reliable and effective processes for manufacturing, quality control, distribution and customer support.

3.6 DEFINITION OF N/P RATIO

The ionic balance within *in vivo*-jetPEI®/nucleic acid complexes is crucial. Indeed, for effective cell entry, the complexes should be cationic. The N/P ratio is a measure of the ionic balance within the complexes and is defined as the number of nitrogen residues of *in vivo*-jetPEI® per nucleic acid phosphate. Approximately one in three nitrogen atoms within the PEI is cationic, therefore electroneutrality of *in vivo*-jetPEI®/nucleic acid complexes is reached at N/P > 2 - 3.

in vivo-jetPEI® is provided as a 150 mM solution (expressed as nitrogen residues). Given that 1 µg of nucleic acid contains 3 nmoles of anionic phosphate, the amount of *in vivo*-jetPEI® to be mixed with DNA in order to obtain a specific N/P ratio is calculated using the following formula:

$$\mu L \text{ of } \textit{in vivo}\text{-jetPEI}^{®} \text{ to be used} = \frac{(\mu g \text{ of DNA} \times 3) \times N/P \text{ ratio}}{150}$$

For *in vivo* nucleic acid delivery experiments, we recommend N/P = 6 - 8. The optimal N/P ratio however should be determined for each new application, animal model and administration route. Please contact the Scientific Support Team for any specific technical request.

3.7 TRADEMARKS

Polyplus-transfection and *in vivo*-jetPEI® are registered trademarks of Polyplus-transfection S.A.

How to cite us: "*in vivo*-jetPEI® (Polyplus-transfection S.A, Illkirch, France)"

3.8 CONTACT INFORMATION

Do you have any technical question regarding your product?

- Website: www.polyplus-transfection.com
- Email: support@polyplus-transfection.com
- Phone: +33 3 90 40 61 87

Contact the friendly Scientific Support team which is composed of highly educated scientists, PhDs and Engineers, with extensive hands on experience in cell culture and transfection. The Scientific Support is dedicated to help our Customers reach their goals by proposing different services such as: protocol optimization, personalized transfection conditions, tailored protocols, etc.

For any administrative question, feel free to contact our administration sales team:

- Reception Phone: +33 3 90 40 61 80
- Fax: +33 3 90 40 61 81
- Addresses:

Polyplus-transfection S.A	Polyplus-transfection Inc.
Bioparc<br>850 Bd S. Brant<br>67400 Illkirch<br>France	1251 Ave of the Americas<br>34th fl.<br>New-York - NY 10020<br>United States

Please note that the Polyplus-transfection support is available by phone from 9:00 am to 5:00 pm CEST.
